# Photokinetics of Mixtures of Independent Photoreactions

**DOI:** 10.3390/molecules30204122

**Published:** 2025-10-17

**Authors:** Mounir Maafi

**Affiliations:** Leicester School of Pharmacy, De Montfort University, The Gateway, Leicester LE1 9BH, UK; mmaafi@dmu.ac.uk

**Keywords:** Φ-order kinetics, photokinetics, photothermal reaction, multicomponent systems, mixtures, quantum yield, sunscreens, photochromes, phytochromes, drugs, monochromatic light, polychromatic light

## Abstract

The photokinetic behavior of concomitant and independent photo- and photothermal reactions exposed to monochromatic or polychromatic irradiation, has not yet been described in photochemistry literature. The occurrence of such mixtures is reported in a wide range of fields, from living species to technologically designed devices. To address the lack of investigative tools that facilitate better understanding, quantification, and control of such parallel-reaction systems, a new holistic approach is proposed in the present study. It contributes to an effort dedicated to rationalizing photokinetics along the same criteria required for thermal kinetics. The methodology builds on a previously introduced general explicit integrated rate-law formula for single-reaction systems (whose integro-differential rate-equation is not solvable). The extension of its field of applicability to multi-component photoreactive mixtures is demonstrated in the present paper. For this purpose, a large number of combinations of both photo- and photothermal individual reactions, possessing distinctly different features, were studied in binary and ternary mixtures. The data of reactions/mixtures were generated by a fourth-order Runge–Kutta numerical integration. An excellent fitting of the species’ kinetic traces by the adapted explicit formula was obtained for all mixtures. Also, the quantification of the effects of the variation in the initial concentration of one component of the mixture, and/or the presence of inert spectator molecules in the reactor, was successfully performed. The investigative photokinetic tools proposed here are shown to be handy, efficient, and useful. The findings of the present study are also thought to expand the application possibilities of reactive photothermal systems in mixtures.

## 1. Introduction

The occurrence of two or more photo- and/or photothermal reactions, concomitantly present and independently reacting inside one reactor, is a common situation in both nature and applied technology. Living plants own a repertoire of stilbene molecules that react simultaneously to light [1]. Nowadays, more medicinal drugs are formulated together within the development of new drug combination therapy treatments (i.e., formulations containing several active pharmaceutical ingredients or multi-API drug formulations). For such drugs, the photostability of the active ingredients’ parallel reactions must be considered [2,3,4]. Parallel-reaction systems also have importance for light-triggered drug release [5,6] and Photobiomodulation [7]. This is, likewise, a classical case for sunscreen cosmetic formulations when the latter involves more than one filter-molecule [8,9,10]. In general, drug formulations contain both active principal ingredient(s) and a variety of excipients that might all concurrently degrade upon direct-light exposure [11].

Running several independent reactions simultaneously can have several potential benefits. In green chemistry, this method might be envisaged to allow saving organic solvents, time, and energy in producing a set of end-products in a one-pot process [12,13]. There is also a merit to such photochemistry for industrial processes, which are currently an intensive subject of research, which otherwise has excellent perspectives for future developments in circular economy [14]. A similar endeavor is prospected for environmental issues as wastewater treatment [15], photocatalysis strategies [16,17,18], and soft matter materials [19]. Recently, a combination of photochromic molecules was employed as part of a single formulation [20], and such combinations make part of some commercially available photochromic ophthalmic lenses [21]. Other examples are found in photocatalytic degradation of two or more species [22], in synthetic photochemistry involving reagents coexisting with the main molecular reactive system of interest [23,24], such as molecular motors [25], nanosystems [26], photoresponsive supramolecular chemistry [27], photodegradation of food products [28], and in a variety of technologies using light-responsive soft matter [29]. Nonetheless, the instances of systems involving more than one photoreactive species extend the non-exhaustive list of cases reported here.

The assessment of such photoreactive systems, for degradation or for efficiency, requires using photokinetic methods and metrics. Unfortunately, published investigations on the photokinetics of simultaneous photoreaction systems are relatively scarce. More importantly, the mathematical kinetic description of multi-concurrent photoreaction systems does not seem to have, thus far, been formalized in the literature [30,31,32]. Most often, the kinetic data of such mixtures was treated by thermal kinetic equations, such as first-order kinetics [2,3,33,34,35], and second-order kinetics [22,36]. The selection of the reaction kinetic order, which would be finally assigned to the reaction at hand, was generally achieved by the same procedure. That is, the reaction order was assigned on the basis of which among the 0th-, first-, and second-order formulae exhibits the highest correlation coefficient value with the reaction data (via the line of best fit). This strategy is, in fact, equally applied for individual reactions (when not involved in a mixture) [32].

For instance, a binary mixture has taken 0th-order kinetics [37], a first-order (mono-exponential) form [2,3,33,37,38,39], or a second-order formulation for mixtures under direct photolysis [22,40,41] or undergoing photocatalytic degradation [36,42]. In these studies, the quantification of the reactions has been performed by several means, such as the half-life time [43], a percentage decrease over a set reaction time [44,45], the rate-constant of the first-order kinetics, approximated initial rates, or by the so-called light screening factor ($s_{\lambda }=(1-10^{-A_{mix}^{\lambda }})/A_{mix}^{\lambda }$) [46,47]. A competitive kinetic method was applied to the simultaneous photodegradation of four phenolic aldehydes under polychromatic light. In this case, a rate expression assuming a constant quantum yield and the absorbance of only the reactant (labeled B), $r_{B}=-\;\Phi_{B}\sum A_{B,i}\;I_{i}$, [48], was used.

Mauser and co-authors have proposed mathematical solutions for the parallel reactions system using a Jacobi matrix method [49]. The method works well when reactions are modeled as mono-exponential patterns, but the cases of combined non-linear photochemical reactions’ rate-laws were not described.

Other methods might have been suggested [50,51,52] for the purpose of describing mixtures of independent photothermal reactions, but these approaches only deal with linear systems useful for thermal reactions, do not consider photo- or photothermal reactions, and do not involve the type of non-linearity induced by the photokinetic factor into the differential equations, the latter being an inherent part of the mathematical description adopted for photochemical systems. Accordingly, such methods are not suitable for the problem mixtures of photoactive components and are, therefore, not reviewed further due to a lack of relevance to the present study.

As a matter of fact, there are, thus far, neither standardized general methods to deal with such a photokinetic problem nor consistent procedures to quantify and assess the photokinetics of mixtures.

The recent advent of a general explicit formula of the integrated rate-law has allowed the description and the complete photokinetic analysis of photo- and photothermal (individual) reactions subjected to either mono- or polychromatic/collimated light. It has fostered the rationalization of photokinetics by simple mathematical tools [53,54,55]. The photoreactions were therein found to obey Φ-order kinetics irrespective of both the overall photoreaction mechanism in play (that might include overlapping thermal reaction-steps), and the variability of the quantum yield with excitation wavelength [55,56]. The Φ-order kinetics is specific to photoreactions under non-isosbestic irradiation. Its model equation is characterized by a particular function, including a decimal logarithm whose argument contains an exponential function (vide infra the photochemical part of Equation (7)). This equation is the first to have been analytically derived in photokinetics [57], with the features of Φ-order kinetics having been previously reviewed [53,58] and contrasted with the properties of the most common models of thermal reaction kinetic.

As stipulated above, explicit integrated rate-laws have not, thus far, been proposed for mixtures involving different reaction systems. Also, it is not known whether the behavior of the individual reactions in the mixture will be characterized by Φ-order kinetics (as the individual photothermal reactions do). An overall analysis indicates that there are at least two arguments that make any prediction of the type (or order) of such kinetics very difficult. On one hand, none of the mixture’s species individual integro-differential rate-laws is analytically solvable. This means that we lack a template for the integrated rate-law describing the kinetic behaviors of mixture-species’ traces. On the other hand, the mixture photokinetics is equivalent to that of one reaction occurring in the presence of a time-variable absorbance of another reaction (i.e., the reactant and products of the other concomitant photoreactive systems). Such a variation in the absorbance might either be monotonically increasing or decreasing or undergoing a variable shape with time. The absorbances and rates of the reactive systems being variable with time, hence, they variably affect the concomitant reactions unfolding through time inside the reactor.

The present investigation attempts to bring a standardization of mixture kinetics via a rational and a mathematical description of concurrent/independent photothermal reactions, and their respective behaviors under various conditions. A set of metrics to assess their reactivity is proposed. Overall, the field of photokinetics will benefit from a standardized description of the mixtures’ kinetics.

## 2. Results and Discussion

### 2.1. General Considerations

The results presented hereafter for reactors involving two or more independent photoreactions, builds its structure on those previously obtained in the photokinetics study of individual reactions driven by either monochromatic or polychromatic light [53,55,57]. Accordingly, the characteristics and features of the individual reactions that we will consider in the present work are supposed to be determined by the methods and procedures established in those previous studies and will not be further discussed here. In this context, it is relevant to emphasize that, whenever possible, separate studies must be conducted on the individual reactions of a mixture before a combination study. Such a characterization of the individual reactions is a prerequisite for numerical simulation of the mixture and predictive modeling. Otherwise, when the individual reactions cannot be studied separately, their mixture can still be characterized (vide infra Equation (9)). However, only the monitoring of the concentration traces (for instance, those reactants of the individual reactions involved in the mixture) is useful to confirm whether the mixture components (two or more photo- and/or photothermal reactions) are independent (vide infra Section 2.7.1).

The kinetic parameters of each individual reaction (Φ and k for each reaction-step, and ε of each species) can be determined by employing the previously set general elucidation method [55] when the kinetic data are obtained for a single reaction in the reactor, that is monitored under monochromatic (non-isosbestic) irradiation, and whose photothermal mechanism is known (the general elucidation method solves the identifiability issue).

Therefore, intrinsic features (i.e., absorption coefficients and photochemical quantum yields and their variations with wavelength) are supposed to be known for all species of each individual reaction making up the mixtures of interest to the present study (obtained from the elucidation method). The extrinsic features, controlled by the experimentalist, are also supposedly known (these include the initial concentrations of the reactants, $C_{X,\chi }(0)$, the wavelength of the monochromatic beam at which the sample is exposed, $\lambda_{irr}$, the incident radiation intensity at every $\lambda_{irr}$, $P_{0}^{\lambda_{irr}}$, the optical path length of the excitation beam within the sample, $l_{irr}$, the optical path length of the monitoring light from the spectrophotometer, $l_{obs}$, and both the irradiated area, $S_{irr}$, and volume, $V_{irr}$, of the sample).

**Scheme 1:**
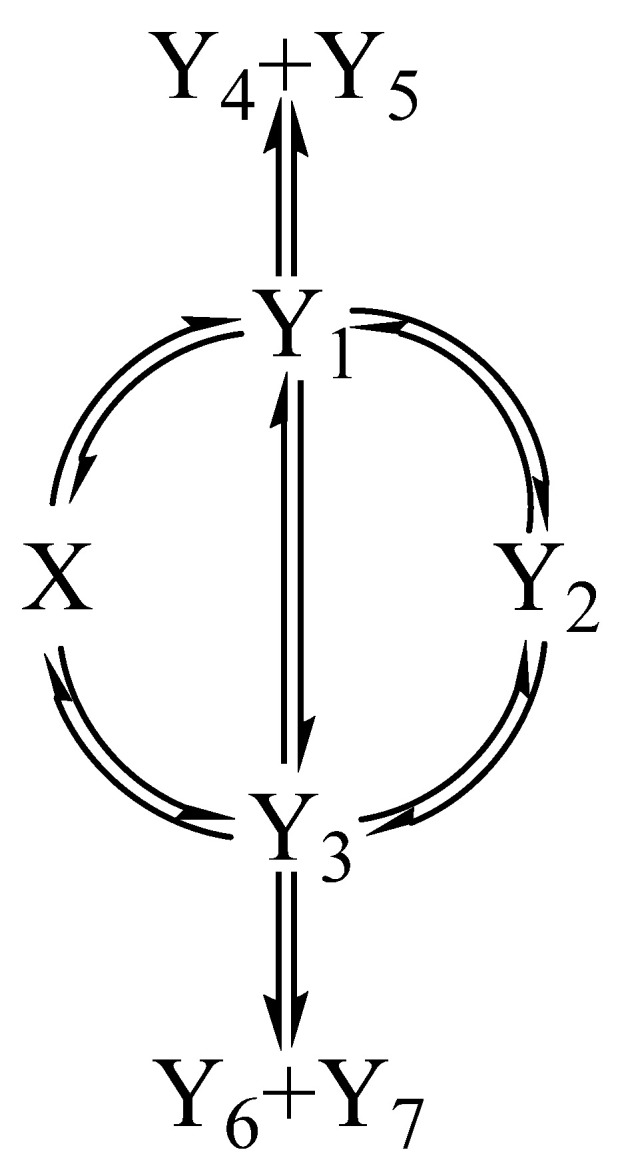
Mechanism of the two $X_{\chi }Y_{7,\chi }(14\Phi )$ photoreactions in the binary mixture (χ=I or II).

The reactions selected for the mixtures belong to the Φ-shaped mechanism (Scheme 1) encompassing eight species that may be interconnected by up to 28 reaction-steps [53,55]. As such, Scheme 1 covers a large set of the known photoreactions’ mechanisms. The reactions are labeled as $X_{\chi }Y_{n_{sp}-1,\chi }(n_{\Phi ,\chi }\Phi ,n_{∆,\chi }k)$ (see definitions in Section 2.4).

Studying binary and ternary mixtures is assumed to be sufficient for practical purposes, so that the concepts and/or the reaction properties verified for these mixtures, should most likely also be valid for mixtures involving more than three components.

The data used to simulate such combination systems are provided by a numerical integration method (NIMs). The consistency of this approach is justified by a number of arguments, which include the following: (i) The acknowledged usage of NIMs to map out experimental data of single or multi-photoreactions with the aim to attempt to elucidate/solve the kinetics at hand [49,59]. (ii) Photokinetic data generated by an NIM has served the development of semi-empirical equations proven to be extremely well verified experimentally [60,61,62,63,64]. (iii) NIMs offer flexibility, versatility, and open vast avenues for a variety of simulations which are not easily accessible experimentally. (vi) They also provide easy access to the data of both the medium (e.g., the total absorbance) and that of the $n_{sp,tot}$ species present in the reactor (i.e., their individual concentration traces). (v) NIMs are time saving and cost-effective as they allow us to study a relatively large number of mixtures. (vi) The usage of NIMs is standard in the wider subject of kinetics, and (vii) NIMs data can be obtained with high accuracy (i.e., the difference with analytically derived integrated rate-laws are very small to negligible, and are well below experimental errors).

The choice of using RK4-NIM is sustained by its known good performance among numerical integration methods [65,66]. Hence, using RK4-NIM for the exploration of photosystems’ photokinetics becomes an interesting, adequate, accurate, and efficient means to describe their behaviors in contrasting situations [55,58].

From a reactivity viewpoint, the modeling proposed here assumes that there are no cross-thermal and/or photochemical reactions between the species of the different components of the mixture (i.e., the different reactions of the mixture) present in the reactor, and no side reactions (e.g., oxidation from the environmental oxygen). Furthermore, the RK-simulations do not consider the occurrence of interference due to photophysical processes (excimer, exciplex, quenching…etc.) between the species present in the mixture.

### 2.2. The System of Fundamental Rate-Law Equations

Each species $Y_{j,\chi }$ in the reactor, amongst those belonging to a binary or a ternary mixture of independent reactive systems ($n_{mix}$), is described by a specific integro-differential equation (Equation (1), expressing the variation in the species’ concentrations, C, with time, t, which are given in units M and s, respectively). For harmonization purposes, the notations, symbols, and quantities’ meanings adopted here bear the same definitions as previously set [55]. The index χ in Equation (1) refers to the specific reactive system within either the binary (i.e., two systems χ=I or II) or the ternary (i.e., three systems, χ=I, II, or III) mixtures. The system of differential equations that describe the reactor at hand would involve the systems of integro-differential equations (Equation (1)) given by the rates $r_{Y_{j,I}}^{Lp,∆\lambda ,T,n_{mix}}(t)$ and $r_{Y_{j,II}}^{Lp,∆\lambda ,T,n_{mix}}(t)$ for a binary, and $r_{Y_{j,I}}^{Lp,∆\lambda ,T,n_{mix}}(t)$, $r_{Y_{j,II}}^{Lp,∆\lambda ,T,n_{mix}}(t),$ and $r_{Y_{j,III}}^{Lp,∆\lambda ,T,n_{mix}}(t)$ for a ternary mixture (r is expressed in $Ms^{-1}$). The rate-law is written here for the case where the mixture is maintained at a constant temperature, T, and the irradiation light is performed with a lamp, *Lp*, whose polychromatic beam spans a ∆λ wavelength region situated between $\lambda_{a}$ and $\lambda_{b}$ ($∆\lambda =\lambda_{a}-\lambda_{b}$, in nm). The photochemical and thermal reaction-steps starting at species $Y_{j,\chi }$ are indicated by the dimensionless photochemical quantum yield at wavelength $\lambda_{irr}$, $\Phi_{Y_{j,\chi }\;⟶\;Y_{j^{\prime ,\chi }}}^{\lambda_{irr}}$, and the rate-constant $k_{Y_{j,\chi }\;⟶\;Y_{j^{\prime },\chi }}^{∆}$ (expressed in $s^{-1}$), respectively (with indices j and $j^{\prime }$ being necessarily of different values). Similarly, the respective parameters are written for the reaction-steps ending at that species, as $\Phi_{Y_{j^{\prime },\chi }\;⟶\;Y_{j,\chi }}^{\lambda_{irr}}$ and $k_{Y_{j^{\prime },\chi }\;⟶\;Y_{j,\chi }}^{∆}$. $n_{\Phi ,j,\chi }$ and $n_{∆,j,\chi }$ correspond, respectively, to the numbers of photochemical and thermal reaction-steps starting or ending at $Y_{j,\chi }$.

(1) $$r_{Y_{j,\chi }}^{Lp,∆\lambda ,T,n_{mix}}\left(t\right)=\pm \frac{dC_{Y_{j,\chi }}^{Lp,∆\lambda ,T,n_{mix}}\left(t\right)}{dt}\;=\int_{\lambda_{a}}^{\lambda_{b}}\left(\sum_{j^{\prime };\;j^{\prime }\neq j}^{n_{\Phi ,j,\chi }}\left(-\Phi_{Y_{j,\chi }\;⟶\;Y_{j^{\prime ,\chi }}}^{\lambda_{irr}}\;P_{a_{Y_{j,\chi }}}^{\lambda_{irr}}\left(t\right)+\Phi_{Y_{j^{\prime },\chi }\;⟶\;Y_{j,\chi }}^{\lambda_{irr}}\;P_{a_{Y_{j^{\prime },\chi }}}^{\lambda_{irr}}\left(t\right)\right)\right)\;d\lambda \;\;+\sum_{j^{\prime };\;j^{\prime }\neq j}^{n_{∆,j,\chi }}\left(-k_{Y_{j,\chi }\;⟶\;Y_{j^{\prime },\chi }}^{∆}\;C_{Y_{j,\chi }}^{Lp,∆\lambda ,T,n_{mix}}\left(t\right)+k_{Y_{j^{\prime },\chi }\;⟶\;Y_{j,\chi }}^{∆}\;C_{Y_{j^{\prime },\chi }}^{Lp,∆\lambda ,T,n_{mix}}\left(t\right)\right)\;$$

The mathematical formulation of the fraction of the light absorbed by a given species in the mixture (expressed in $einstein\;{dm}^{-3}s^{-1}$), at a given time t and wavelength $\lambda_{irr}$, is

(2) $$P_{a_{Y_{j,\chi \;or\;j^{\prime },\chi }}}^{\lambda_{irr}}\left(t\right)=\frac{A_{Y_{j,\chi \;or\;j^{\prime },\chi }}^{\lambda_{irr},T,n_{mix}}\left(t\right)}{A_{tot}^{\lambda_{irr}T,n_{mix}}\left(t\right)}\;P_{0}^{\lambda_{irr}}\;\left(1-10^{-A_{tot}^{\lambda_{irr},T,n_{mix}}\left(t\right)}\right)\;=A_{Y_{j,\chi \;or\;j^{\prime },\chi }}^{\lambda_{irr},T,n_{mix}}\left(t\right)\;P_{0}^{\lambda_{irr}}\;PKF^{\lambda_{irr},T,n_{mix}}\left(t\right)$$

$PKF^{\lambda_{irr},T,n_{mix}}(t)$ in Equation (2), is the dimensionless photokinetic factor at $\lambda_{irr}$. The total absorbance, $A_{tot}^{\lambda_{irr}T,n_{mix}}\left(t\right)$, corresponds to a sum of the absorbancies, $A_{Y_{j,\chi }}^{\lambda_{irr},T,n_{mix}}$, of all species absorbing at the considered wavelength $\lambda_{irr}$ and time t, as given by Equation (3) (i.e., including each reactive system within the reactor, i.e., reactions I,II, and/or III, that encompasses $n_{sp,\chi }$ species in its mechanism).

(3) $$A_{tot}^{\lambda_{irr}T,n_{mix}}\left(t\right)=\sum_{j=0}^{n_{sp,I}}A_{Y_{j,I}}^{\lambda_{irr},T,n_{mix}}(t)+\sum_{j=0}^{n_{sp,II}}A_{Y_{j,II}}^{\lambda_{irr},T,n_{mix}}(t)+\sum_{j=0}^{n_{sp,III}}A_{Y_{j,III}}^{\lambda_{irr},T,n_{mix}}(t)$$

The validity of Equation (2) imposes that absorbances of the individual species in the reactor, that absorb at $\lambda_{irr}$ ($A_{Y_{j,\chi \;or\;j^{\prime },\chi }}^{\lambda_{irr},T,n_{mix}}\left(t\right)$), each have a value that falls within the linearity range of the corresponding species’ calibration graph (it was suggested [53,55] that this condition should in principle be satisfied if the value of $A_{tot}^{\lambda_{irr}T,n_{mix}}\left(t\right)$ does not exceed the value 0.5 throughout the reaction time).

The rate-laws of the species of the multi-reaction mixtures (Equation (1)) are all coupled together by two factors, namely, at given $\lambda_{irr}$, the same values for $P_{0}^{\lambda_{irr}}$ and $A_{tot}^{\lambda_{irr}T,n_{mix}}$ (or $P_{0}^{\lambda_{irr}}$ and $PKF^{\lambda_{irr},T,n_{mix}}\left(t\right)$). The coupling through $PKF^{\lambda_{irr},T,n_{mix}}\left(t\right)$ reflects a competition between the photoreactive species present at time t, for the light, at $\lambda_{irr}$, entering the reactor.

The system of integro-differential equations describing a given reactor, Equation (1), is not possibly analytically solvable due to the non-linear character of the photokinetic factor. The latter is present in each of the individual differential equations making up the mathematical system. The same hurdle also impedes reaching analytical integrations of the rate-laws in the case of individual photothermal reaction considered under collimated monochromatic irradiation [58] (the exception being the primary photoreaction whose photoproduct is transparent to the monochromatic irradiation [57,58]). The difficulty of integration is even more acute if the functions describing the wavelength-variation in the incident radiation, absorption, and quantum yield are not known a priori for each species interacting with the excitation light.

The equations of the type of Equation (1) have not benefited from the standard methods applied to solve some integro-differential equations [67,68], except for a single recently published example [69] employing high-order positivity-preserving approximation schemes for photoreactions’ integro-differential equations using numerical methods. However, in the latter study, the temporal evolutions of the concentrations were assumed to obey first-order kinetics (i.e., the rate does not involve the non-linear PKF, but rather dC/dt=∑k C).

To overcome the difficulty of analyzing and computing Equation (1) rate-law systems, an effective way is to replace the integral over λ in Equation (1) by a summation at a 1 nm step [53,55,64,66]. Such a transformation yields Equation (4), that can readily be evaluated by numerical integration methods.

(4) $$r_{Y_{j,\chi }}^{Lp,∆\lambda ,T,n_{mix}}\left(t\right)=\pm \frac{dC_{Y_{j,\chi }}^{Lp,∆\lambda ,T,n_{mix}}\left(t\right)}{dt}\;=\sum_{\lambda_{irr}=\lambda_{a}}^{\lambda_{b}}\left(\sum_{j^{\prime };\;j^{\prime }\neq j}^{n_{\Phi ,j,\chi }}\left(-\Phi_{Y_{j,\chi }\;⟶\;Y_{j^{\prime ,\chi }}}^{\lambda_{irr}}\;P_{a_{Y_{j,\chi }}}^{\lambda_{irr}}\left(t\right)+\Phi_{Y_{j^{\prime },\chi }\;⟶\;Y_{j,\chi }}^{\lambda_{irr}}\;P_{a_{Y_{j^{\prime },\chi }}}^{\lambda_{irr}}\left(t\right)\right)\right)\;+\sum_{j^{\prime };\;j^{\prime }\neq j}^{n_{∆,j,\chi }}\left(-k_{Y_{j,\chi }\;⟶\;Y_{j^{\prime },\chi }}^{∆}\;C_{Y_{j,\chi }}^{Lp,∆\lambda ,T,n_{mix}}\left(t\right)+k_{Y_{j^{\prime },\chi }\;⟶\;Y_{j,\chi }}^{∆}\;C_{Y_{j\prime ,\chi }}^{Lp,∆\lambda ,T,n_{mix}}\left(t\right)\right)$$

In the case of an irradiation with a monochromatic light, Equation (4) loses the summation over λ, and the labeling $Lp,∆\lambda ,T,n_{mix}$ is changed to $\lambda_{irr},T,n_{mix}$. For purely photochemical reactions, the rate-constants of the thermal reaction-steps, in Equation (4), are given zero values ($k^{∆}=0$). Whereas $P_{0}^{\lambda_{irr}}=0$ is implemented in Equation (4) for purely thermal reactions.

In the present work, the fourth-order Runge–Kutta (RK4) method is used to evaluate the photokinetics of binary or ternary systems. The RK-simulated traces are not intended here for the determination of the reactions’ features and parameters by fitting them to experimental data, as usually employed in photokinetic literature [49,66] (the latter approach is most likely not capable of solving the identifiability problems). Instead, here, such RK-calculated traces are used to test the validity of an explicit integrated rate-law (vide infra Equation (7)). It also serves to describe this equation’s ability to predict and to quantify the kinetic behavior of the reactive systems involved in the investigated mixture. The explicit formula hence intends to fill a gap in the knowledge, as no such mixture specific explicit equations exist in the literature today [21,38,70].

The explicit equation proposed in the present work (vide infra Equation (7)), is expected to be capable of fitting the species’ kinetic traces that are obtained from a mixture of independent photo- and/or photothermal reactions exposed to either mono- or polychromatic light irradiation, and irrespective of the mechanisms governing the individual reactions involved in the mixture. Once validated, the explicit formula is to be treated as any analytically derived integrated rate-law when applied to experimental data.

The initial reactant-rate of the concentration trace for a reaction starting from a thermally stable reactant (j=0 and $Y_{0,\chi }=X_{\chi }$), is worked out from Equation (4), as

(5) $$Theo:\;r_{X_{\chi }}^{Lp,∆\lambda ,T,n_{mix}}\left(0\right)=Theo:\;r_{0,X_{\chi }}^{Lp,∆\lambda ,T,n_{mix}}\;=-\sum_{\lambda_{irr}=\lambda_{a}}^{\lambda_{b}}\left(\sum_{j^{\prime };\;j^{\prime }\neq 0}^{n_{\Phi ,0,\chi }}\Phi_{X_{\chi }\;⟶\;Y_{j^{\prime ,\chi }}}^{\lambda_{irr}}\;P_{a_{Y_{0,\chi }}}^{\lambda_{irr}}\left(0\right)\right)$$

It is also useful to define the theoretical formula for the initial rate of the total absorbance trace ($r_{0,A}^{Lp,∆\lambda ,T,n_{mix}/\lambda_{obs}}$). The reactive medium is observed at $\lambda_{obs}$ and the absorbancies are measured across the optical path length $l_{obs}$. The observation ($\lambda_{obs}$ and $l_{obs}$) and irradiation ($\lambda_{irr}$ and $l_{irr}$) features are not, respectively, necessarily equal ($l_{obs}$ and $l_{irr}$, are both expressed in cm). In the case we assume that the reactants of the individual reactions composing the mixture are thermally stable, and the mixture reactions obey sub-mechanisms of the Φ-shaped mechanism (Scheme 1, where the reactants undergo, at most, a divergent reaction, as $Y_{1,\chi }\leftarrow X_{\chi }\to Y_{3,\chi }$), the $r_{0,A}^{Lp,∆\lambda ,T,n_{mix}/\lambda_{obs}}$ expression takes the form

(6) $$\;Theo:\;r_{0,A}^{Lp,∆\lambda ,T,n_{mix}/\lambda_{obs}}=\;\;l_{obs}\sum_{\chi =I}^{III}\left(\varepsilon_{X_{\chi }}^{\lambda_{obs}}\;Theo:\;r_{0,X_{\chi }}^{Lp,∆\lambda ,T,n_{mix}}+\varepsilon_{Y_{1,\chi }}^{\lambda_{obs}}\;Theo:\;r_{0,Y_{1,\chi }}^{Lp,∆\lambda ,T,n_{mix}}+\varepsilon_{Y_{3,\chi }}^{\lambda_{obs}}\;Theo:\;r_{0,Y_{3,\chi }}^{Lp,∆\lambda ,T,n_{mix}}\right)$$

where ε is the absorptivity of the species at the indicated wavelength (expressed in $M^{-1}{cm}^{-1}$).

### 2.3. The Model Integrated Rate-Law

A qualitative assessment of published experimental data [2,3,20,38,39,65,71,72] does not suggest strong differences between the overall patterns of the kinetic traces observed for mixtures of photothermal reactions and those obtained for a reaction alone in the reactor.

The conjecture is, hence, that the superposition of the kinetics in multi-component reactive systems does not alter the basic character of the kinetic behavior of the individual species, which should still obey Φ-order kinetics. Therefore, the overall formulation of the explicit formula describing photothermal reactions’ kinetics [55] should also be the dedicated format of explicit integrated rate-laws depicting the species traces when evolving within multi-component mixtures (but including the relevant amendments relative to a mixture of reactions). This conjecture can perhaps be supported by previously studied cases, where spectator molecules (SPMs) were present in the reactor of individual photo- and photothermal reactions considered under mono- and polychromatic light. Here, the SPMs absorbance increased but the initial concentration of the reactant remained constant from one experiment to the other [53,55]. Therefore, when the concentrations of the reaction species vary with time, under irradiation, the amount of light available to each species of the reaction (in the presence of SPM) is also variable with time. This might be analogous to the case of multi-component systems where the various species in the reactor are all receiving variable amounts of light during the progress of the reactions with time. Furthermore, since the individual reactions of the mixture are independent from each other (i.e., no cross or side reactions, or interactions between them), the only variable is the amount of light received by each species at reaction time t, and therefore, the general kinetic pattern of a given reaction should be independent of whether the reaction is isolated or part of a mixture.

Hence, the temporal evolution of the concentration of an individual species $Y_{j,\chi }$, belonging to any of the reactive systems (I, II or III) present in the reactor, is mapped out by the following general equation (Equation (7)), that stands for the integrated rate-law of that species.

(7) $$C_{Y_{j,\chi }}^{Lp,∆\lambda ,T,n_{mix}}\left(t\right)=\omega_{j,\chi }^{\infty }+\sum_{i=1}^{i_{\Phi ,j,\chi }}\omega_{ij,\chi }^{\Phi }\;Log(1+{cc}_{j,\chi }^{\Phi }\;e^{-k_{ij,\chi }^{\Phi }\;t})\;+\sum_{i=1}^{i_{∆,j,\chi }}\omega_{ij,\chi }^{∆}\;e^{-k_{ij,\chi }^{∆}\;t}\;$$

Equation (7) merges the Φ-order ($\omega \;Log\left(1+cc\;e^{-\;k\;t}\right)$) and the first-order ($\omega \;e^{-\;k\;t}$) characters that govern the photochemical and thermal reaction-steps, respectively. Either $\omega^{\Phi }$ or $\omega^{∆}$ take zero values when the considered $Y_{j,\chi }$ does not link to either a photochemical or a thermal reaction-step, respectively.

The numbers $i_{\Phi ,j,\chi }$ and $i_{∆,j,\chi }$ (in Equation (7)) are practically determined from the fitting process but they must be, at most, equal to $n_{\Phi ,j,\chi }$ and $n_{∆,j,\chi }$, respectively.

The remaining coefficients of Equation (5) ($\omega_{j,\chi }^{\infty }$, $\omega_{ij,\chi }^{\Phi }$, $\omega_{ij,\chi }^{∆}$, ${cc}_{j,\chi }^{\Phi }$, $k_{ij,\chi }^{\Phi }$ and $k_{ij,\chi }^{∆}$), are also obtained from the fitting process. $\omega_{j,\chi }^{\infty }$ corresponds to the concentration of species $Y_{j,\chi }$ at the end of its reaction (i.e., at t=∞, either complete depletion (i.e., $\omega_{j,\chi }^{\infty }=0$), or an equilibrium or photostationary state (i.e., $\omega_{j,\chi }^{\infty }\neq 0$)). The pre-logarithmic and pre-exponential coefficients, ω and cc, are weighing factors for the corresponding $i^{th}$ kinetic regime of the considered species $Y_{j,\chi }$. Each of the ks corresponds to the positive rate-constant of the $i^{th}$ kinetic regime. Even though explicit formulae of these coefficients are not yet known, Equation (7) is still very useful for quantification purposes of the kinetics at hand, as shall be seen in the following sections.

Equation (7) is the first in the photochemistry literature to be proposed for multi-component systems of independent photo- and/or photothermal reactions under poly- or monochromatic light irradiation, irrespective of the photo- or photothermal mechanisms governing the involved reactions of the mixture.

The justification of the mathematical formulation taken by Equation (7) was discussed in previous work [53,54,55]. It stems from a parallel with the thermal reaction kinetics. In fact, the latter is characterized by a linear combination of mono-exponential terms, where the mono-exponential form is analytically derived for the kinetics of the basic unimolecular thermal reaction. An equivalent to the latter situation is observed for the photokinetics of photothermal reactions exposed to monochromatic and isosbestic light. By analogy, the kinetics of complex photochemical reactions under non-isosbestic irradiation (whose rate-laws are not possibly analytically solvable), is predicted to be described by an integrated rate-law composed of a combination of mono-Φ-order terms, the very format that is analytically derived for the unimolecular photoreaction [57,58]. This approach is more consistent than considering a formulation based on a different function than the mono-Φ-order term. In this context, the inappropriateness of replacing the mono-Φ-order term by, for instance, a mono-exponential function has been proven (such a swap results in an improper fitting of the unimolecular photoreaction traces [58]).

Differentiation of Equation (7) at the initial time leads to the expression of the initial rate value of $Y_{j,\chi }$ concentration trace, as

(8) $$Fit:\;r_{Y_{j,\chi }}^{Lp,∆\lambda ,T,n_{mix}}\left(0\right)=Fit:\;r_{0,Y_{j,\chi }}^{Lp,∆\lambda ,T,n_{mix}}=\;-\frac{1}{\ln\left(10\right)}\;\sum_{i=1}^{i_{\Phi ,j,\chi }}\omega_{ij,\chi }^{\Phi }\;k_{ij,\chi }^{\Phi }\frac{{cc}_{j,\chi }^{\Phi }}{1+{cc}_{j,\chi }^{\Phi }}-\sum_{i=1}^{i_{∆,j,\chi }}\omega_{ij,\chi }^{∆}\;k_{ij,\chi }^{∆}\;$$

The total absorbance trace of a multi-component system observed at $\lambda_{obs}$, $A_{tot}^{Lp,∆\lambda ,T,n_{mix}/\lambda_{obs}}\left(t\right)$, is fitted with an equation (Equation (9)) similar to Equation (7), with specific Φ-order ($i_{\Phi ,A}\leq \sum_{\chi =I}^{III}n_{\Phi ,j,\chi }$) and first-order ($i_{∆,A}\leq \sum_{\chi =I}^{III}n_{∆,j,\chi }$) terms. The coefficients of Equation (9) have the same meanings as their counterparts listed for Equation (7).

(9) $$A_{tot}^{Lp,∆\lambda ,T,n_{mix}/\lambda_{obs}}\left(t\right)=\omega_{A}^{\infty }+\sum_{i=1}^{i_{\Phi ,A}}\omega_{i,A}^{\Phi }\;Log(1+{cc}_{A}^{\Phi }\;e^{-k_{i,A}^{\Phi }\;t})\;+\sum_{i=1}^{i_{∆,A}}\omega_{i,A}^{∆}\;e^{-k_{i,A}^{∆}\;t}\;\;$$

The initial rate of the total absorbance trace is obtained from the differentiation of Equation (9), as

(10) $$Fit:\;r_{0,A}^{Lp,∆\lambda ,T,n_{mix}/\lambda_{obs}}\left(0\right)=Fit:\;r_{0,A}^{Lp,∆\lambda ,T,n_{mix}/\lambda_{obs}}\;=-\frac{1}{\ln\left(10\right)}\;\sum_{i=1}^{i_{\Phi ,A}}\omega_{i,A}^{\Phi }\;k_{i,A}^{\Phi }\frac{{cc}_{A}^{\Phi }}{1+{cc}_{A}^{\Phi }}-\sum_{i=1}^{i_{∆,A}}\omega_{i,A}^{∆}\;k_{i,A}^{∆}\;$$

### 2.4. Validation of the Explicit Model Equations

#### 2.4.1. The Good Fitting of the Traces

The consistency of the explicit model equation (Equation (7)) to describe the concentration traces of species belonging to concomitant and independent reactions is tested against various RK-generated traces. The data considered here belong to reactive systems with different characteristics (possessing different intrinsic (Φ,k,ε) and extrinsic ($C_{X,\chi }\left(0\right),\;P_{0},l$) features). The studied multi-reaction systems included various combinations of reactions of different photo- and photothermal mechanisms.

**Figure 1:**
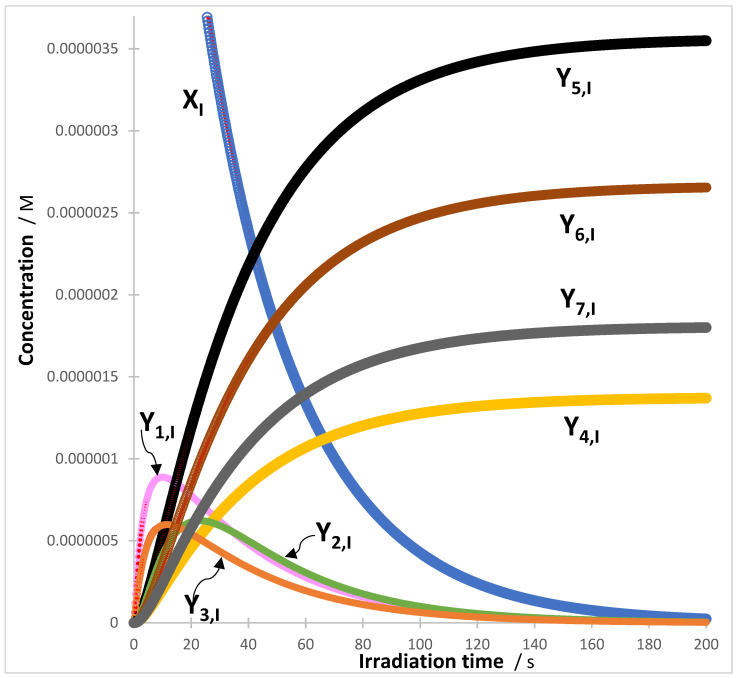
An example of a good fit of the eight reaction’s species RK-traces (circles) by their corresponding Equation (7) (lines) for the first $X_{I}Y_{7,I}(14\Phi )$ reaction of the binary mixture (Scheme 1).

More than 100 RK-traces were RK-generated accordingly and fitted with the appropriate Equations (7) and (9). The goodness of fit was assessed by correlation coefficient values between RK-calculated and Equation (7) (or Equation (9)) data, close to unity (>0.999), low values of both sums of squared errors (SSE < 10^−14^), and the root-mean-square errors (RMSE < 10^−7^). An illustration example is provided by the binary mixture of two (*I* and *II*) different $XY_{7}(14\Phi )$ sequences (Scheme 1), driven by a monochromatic light (see details in Supplementary Information, Section S1). The fitted traces of *Reaction I* are shown in Figure 1.

The traces of the species of both mixture components were fitted by Equation (7) that encompass two or three mono-Φ-order terms (i.e., $i_{\Phi ,j,\chi }\leq 3\leq n_{\Phi ,j,\chi }\leq 4<n_{\Phi ,\chi }=14$, and $i_{∆,j,\chi }=0$ since $\omega_{ij,\chi }^{∆}=0$) because there are no thermal reaction-steps in the reaction mechanisms (Scheme 1). For *Reaction I* (Figure 1), the eight traces share the same values of the three rate-constants of the photochemical-steps ($k_{1j,I}^{\Phi }=0.0288$, $k_{2j,I}^{\Phi }=0.1245$ and $k_{3j,I}^{\Phi }=0.4377\;\;s^{-1}$).

The excellent fitting of the traces accounts for the reliability of Equation (7) to describe the kinetic behavior of the individual species’ concentration traces when the latter are present in a reactor that involves more than one concurrent but independent reaction. These findings demonstrate that the kinetic behavior of an individual species conserves, overall, its Φ-order character when other reactions occur in the reactor, as long as there are no thermal, photophysical, and/or photochemical interferences between the species of the individual reactions (e.g., species of *Reaction I* and those of *Reaction II*, in a binary mixture). The conservation of the Φ-order type of the kinetics sets out an unprecedented result in the area of mapping out photokinetics of multi-component photo- and photothermal reaction systems.

A similar approach can be considered for the fitting of the traces corresponding to the total absorbance of the medium that is measured at a given observation wavelength ($\lambda_{obs}$) and through the monitoring optical path length ($l_{obs}$). The good fit of the RK-data with Equation (9) is both a supplementary confirmation of the validity of the explicit equations, and a valuable tool for the treatment of experimental data.

The excellent correspondence between both Equations (7) and (9) with the RK4-generated traces is an essential condition for the validation of these explicit equations.

#### 2.4.2. The Correspondence of the Initial Rates

A good fitting of the traces by Equations (7) and (9) is a necessary condition. However, there is a need for a quantification metric to further the kinetic analysis.

The reaction rate-constant is the usually preferred metric in thermal kinetics. But this is true only for the simplest reactions. For more complex or extended reactions, the determination of the true values of the rate-constants of the various reaction-steps is not possible. The case of the consecutive mechanism involving two thermal reaction-steps ($X\to Y_{1}\to Y_{2}$) is illustrative of such a difficulty if the absorptivity of the intermediate species is unknown (or when $Y_{1}$ cannot be physically isolated). Here, several values of the set of the three reaction parameters (two rate-constants and the absorptivity of $Y_{1}$), representing the various kinetic solutions, can be found. The introduction of each of these kinetic solutions into the integrated rate-laws of the reaction (which are either mono- or double exponential functions) lead to faithful reproduction of the kinetic traces. A degeneracy of the kinetic solution indicates the occurrence of an identifiability issue [73,74,75,76]. A similar identifiability issue arises for the fitting parameters of Equation (7) when the latter is fitted to the reaction traces of photothermal reactions [53,54,55]. The variety of fitting parameters’ sets of Equation (7) (w, cc and k) to a given trace hampers the singling out of the true values of the rate-constants (of photochemical and thermal processes). As a result, the rate-constants, k, cannot be used for quantification.

Accordingly, it is useful to consider the initial reaction rate ($r_{0}$) as a metric. This quantity is the only one that can directly be calculated by both the numerical method, $RK:\;r_{0}$, (the rate-constant k being inaccessible by this means), and from the fitting parameters (Equations (8) and (10)), irrespective of the identifiability of the fitting parameters [53,54,55] (i.e., a unique value of $r_{0}$ is calculated from Equation (8) (or Equation (10)), whatever the set of the values of the fitting parameters, as long as Equation (7) (or Equation (9)) do fit well with the corresponding trace). Such $r_{0}$ values can also be compared to the ones ($Theo:\;r_{0}$) theoretically obtained from Equations (5) and (6) at the initial time. The coefficients used to calculate theoretical values by Equations (5) and (6), are those which have originally fed the RK-calculation of the multi-component system at hand.

**Figure 2:**
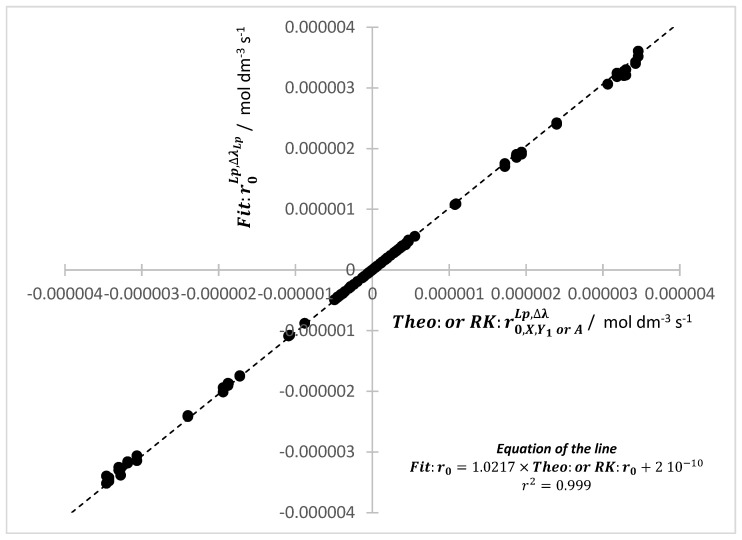
Excellent linear correlation between the values of $Fit:\;r_{0{,Y}_{j,\chi }}^{\lambda_{irr},orLp}$ and both $Theo:\;r_{0{,Y}_{j,\chi }}^{\lambda_{irr},orLp}$ and $RK:\;r_{0{,Y}_{j,\chi }}^{\lambda_{irr},orLp}$ (with j=0,1 or 3) of reactants’ consumptions and their adjacent/immediate products’ formations for various/different binary and ternary reactive systems subjected to both mono- and polychromatic light (# of points 135).

Effectively, the multi-reaction systems simulated in the present study provide good evidence (Figure 2) for the correspondence of these three values of the initial rates of the reactants ($X_{\chi }$) and their immediate photoproducts ($Y_{1,\chi }$ and $Y_{3,\chi }$, according to Scheme 1). The linear trend is characterized by both correlation coefficient and gradient, almost equal to unity, and a very small value of the intercept. The maximum percentage error value recorded between $Theo:\;r_{0}$, $RK:\;r_{0}$, and $Fit:\;r_{0}$ does not exceed 5%.

The fitting and $r_{0}$ features consolidate the validity of Equations (7) and (9). It also demonstrates that testing any new, and not analytically derived, equations against a large number of RK-generated reaction data is a very effective means to decide on the consistency and usefulness of such proposed formulae. This falls in line and extends the overall approach using RK-data to test and validate sets of integrated rate-laws (such as Equations (7) and (9)), as it was previously demonstrated in many cases [53,54,55,56,57,61,62,63,64]. This is a strategy that can be highly recommended for future developments in photokinetics. This approach is also interesting as it considers wavelength variable quantum yields and polychromatic light irradiation.

The ubiquity of quantum yield variability with wavelength [56], raises the importance of this feature for holistic and accurate photokinetic analyses.

### 2.5. Variation in Reaction Rates of a Molecule When Alone and in a Mixture

Performances of the components of mixtures were compared when irradiated alone and when concomitantly reacting in the mixture. The initial reaction rates of isolated reactions were always higher than the respective ones recorded for the same reaction in the mixture. Also, when mixed, the compounds are less reactive than when they are alone. This phenomenon is always true as long as the absorption spectra of the mixture’s components overlap, and the region of overlap is with either the profile of the lamp or the monochromatic light used (i.e., the so-called *OSIA* region [55]). The photokinetic explanation is hence straightforward, that is, the individual photokinetic factors (*PKF*) of the components reacting alone (in separate reactors) have higher values than the ones recorded for when they are present in the mixture, since the total absorbance has a higher value for the mixture (Equation (2)). Therefore, the rate and, by ricochet, the initial reaction rate of each component in the mixture should have a lower value than that of each component reacting alone.

These results are corroborated by the experimental observation of the reactions of six parabens irradiated separately and when considered in a mixture [35]. The pseudo-first-order reaction rate-constant value reduced for each paraben in the mixture compared to their rate-constants when photolyzed alone. Since the same irradiation conditions were used for all experiments (i.e., the same initial incident number of photons received by the reactor), the authors attributed the reactivity slowdown to a lesser amount of incident photons that would be available to each molecule in the mixture compared to the individual reactions of each paraben alone. This idea is quantified by the photokinetic interpretation given above.

### 2.6. Contribution of Spectator Molecules

Often, real mixtures include compounds that do not belong to the photo- or photothermal reactive systems of interest, and which do not thermally or photochemically react. These inert molecules are dubbed the spectator molecules (SPMs) and their number is labeled $n_{SPM}$. They acquire importance in photokinetics when the absorption spectrum of an SPM, over a wavelength region of the light impinging on the reactor, overlaps that of one or more of the photoactive species belonging to the studied reactive systems of the mixture. This means that there is concomitant light absorption by the SPM and the photoactive species, of which the latter must be photoreactive, i.e., their quantum yields must not be equal to zero over the considered wavelength region of irradiation.

In such a case, the SPMs have, a priori, no effect on the thermal reaction-steps, but they will affect the concerned photochemical reaction-steps. Their contribution relates directly to a modification of the expression of the total absorbance $A_{tot}^{\lambda_{irr}T,n_{mix}}\left(t\right)$ (Equation (3)), whose value makes part of the absorbed light formula (Equation (2)). It now must incorporate an additional term corresponding to SPMs (Equation (11)).

(11) $$A_{tot}^{\lambda_{irr}T,n_{mix}}\left(t\right)=\sum_{\chi =I}^{n_{mix}}\sum_{j=0}^{n_{sp,\chi }}A_{Y_{j,\chi }}^{\lambda_{irr},T,n_{mix}}(t)+\sum_{r=1}^{n_{SPM}}A_{{SPM}_{r}}^{\lambda_{irr},T,n_{mix}}$$

The SPMs effectively shield, by absorption, a fraction of the incident light from the photochemical reactive species at the individual $\lambda_{irr}$ over the region where the spectrum of the SPMs, that of the photoreactive systems, and the profile of the light source overlap. An increase in the SPMs absorbance (i.e., increase in their concentrations) would invariably lead to a decrease in the amount of light available to be absorbed by the targeted species in the mixture, which consequently, induces a reduction in reactivity (see Supplementary Information, Section S2). As the photokinetic factor, PKF (Equation (2)), is shared by all the species absorbing at the given set of $\lambda_{irr}$, and the initial concentration of the mixture components are constant (for each separate experiment while $A_{{SPM}_{r}}^{\lambda_{irr},T,n_{mix}}$ increases), the photo-transformation rates of all components of the mixture should proportionally diminish. Hence, for a mixture of $n_{mix}$ reactive systems, it is expected to record both smaller values for the initial rates, and a slowdown of the reactivity of all mixture components with an increasing concentration of the SPMs (that absorb in the same region covered by the light where the component photoactive species also absorb).

**Figure 3:**
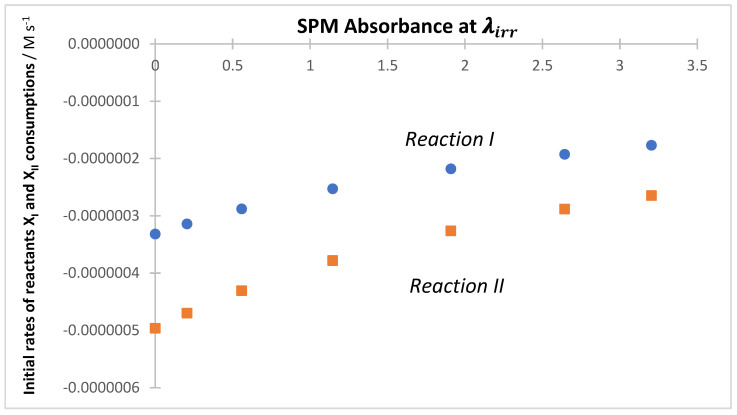
A progressive decrease in both initial rates values of the individual reactions of the binary mixture ($r_{0X_{I}}^{\lambda_{irr}}$ and $r_{0X_{II}}^{\lambda_{irr}}$, as given in Scheme 2) with increasing SPM absorbance at wavelength ($\lambda_{irr}$) of the monochromatic irradiation.

**Scheme 2:**
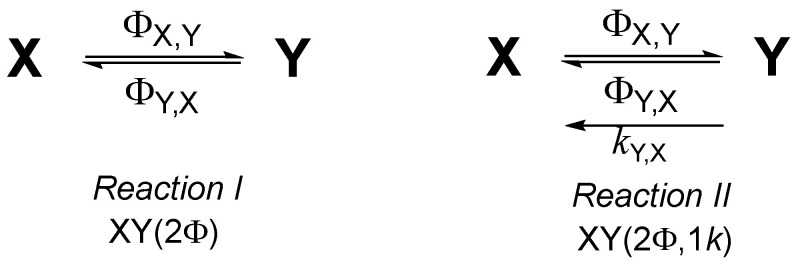
Components of the binary mixture.

The effect of SPMs has been evidenced for individual reactions under both monochromatic [53,63,77] and polychromatic light [54,55]. The variation in the initial reactants’ rate ($r_{0X_{\chi }}^{\lambda_{irr}}\to -0$) with increasing $A_{{SPM}_{r}}^{\lambda_{irr},T,n_{mix}}$ is illustrated in Figure 3 for the binary mixture of Scheme 2.

### 2.7. Initial Concentration Effects on Reactivity

Changing the initial concentration ($C_{X,\chi }(0)$) of one or several reactants of a mixture, would change the coefficients of the rate-law that involve the initial concentration (such as PKF(t)). In principle, a change of $C_{X,\chi }(0)$ does not affect the overall formulation of $C_{X,\chi }(t)$ as given by Equation (4) (supposing no interactions occur between the reactive species of the different reactions of the mixture). It remains important, however, to assess whether the kinetics behavior of the species would still obey Equation (7) (i.e., to be of Φ-order type). This is a significant difference to the case where SPMs are present in the medium (whose (SPMs) concentrations remain constant throughout the time during which the mixture is subjected to irradiation). Conversely, when increasing $C_{X,\chi }(0)$, the amount of light available to each photoreactive species in the mixture becomes variable with time (because it is also variable with the initial concentrations of the reactants, as the absorbances are time dependent).

RK-simulations performed under this condition (various $C_{X,\chi }(0)$) yield smooth kinetic traces for the species. These traces are similar to those observed for the multi-component system at any given initial reactants’ concentrations. We will explore, in the following section, the effects of concentrations in two multi-component systems—one binary and one ternary.

#### 2.7.1. A Binary Mixture Under Monochromatic Light

**Figure 4:**
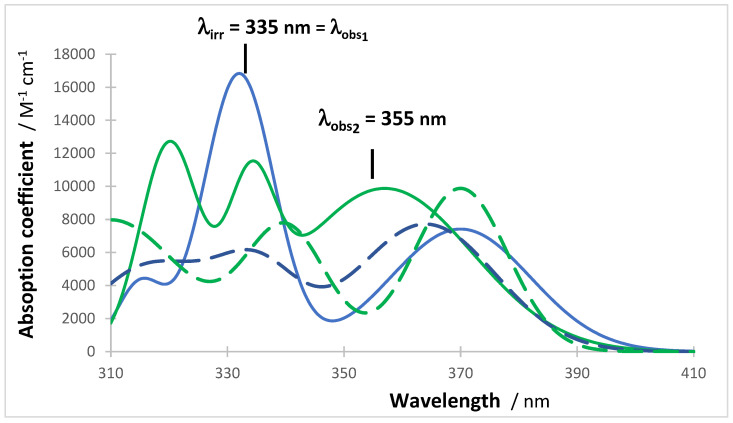
Absorption spectra (ε units) of the reactants (blue) and products (green) of *Reaction I* (plain lines) and *Reaction II* (discontinued lines). Irradiation and observation wavelengths are indicated.

A photo-XY(2Φ) and a photothermal XY(2Φ,1k) reversible reactions (Scheme 2) make up an example of a mixture evolving under monochromatic light. The different absorption spectra of the four species overlap almost completely over 310–410 nm (Figure 4).

Irradiation and observation wavelength ($\lambda_{irr}=\lambda_{obs_{1}}=335\;nm$) and optical path lengths ($l_{irr}=l_{obs}=1.58\;cm$) are selected here, respectively, to be equal. The monochromatic beam ($2.12\times 10^{-6}\;einstein\;dm^{-3}\;s^{-1}$), continuously impinging on the reactor, activates the four species. The quantum yields of *Reaction I* ($\Phi_{X_{I}\;⟶\;Y_{I}}^{335}=0.126$ and $\Phi_{Y_{I}\;⟶\;X_{I}}^{335}=0.152$) have a reverse trend to those of *Reaction II* ($\Phi_{X_{II}\;⟶\;Y_{II}}^{335}=0.136$ and $\Phi_{Y_{II}\;⟶\;X_{II}}^{335}=0.101$).

The rate-constant of the thermal reaction was $k_{Y_{II}\;⟶\;X_{II}}^{∆}=0.0429\;\;s^{-1}$, and the absorptivities (in $M^{-1}{cm}^{-1}$) of the reactants were $\varepsilon_{X_{I}}^{335}=14,919.5$ and $\varepsilon_{X_{II}}^{335}=6076.95$, and the products of those were $\varepsilon_{Y_{I}}^{335}=11,471.04$ and $\varepsilon_{Y_{II}}^{335}=6660.44$.

**Figure 5:**
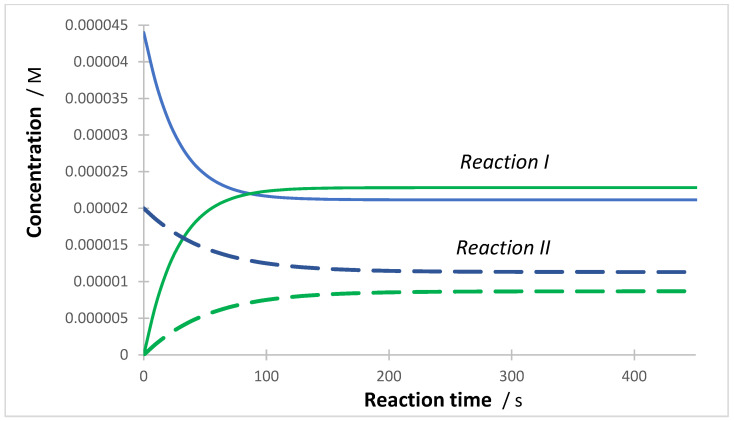
Concentration kinetic traces of the reactants (blue) and products (green) of *Reaction I* (plain lines) and *Reaction II* (discontinued lines). The initial concentrations are $C_{X,I}^{335,T}\left(0\right)=4.4\times 10^{-5}\;M$ and $C_{X,II}^{335,T}\left(0\right)=2\times 10^{-5}\;M$.

Both reactions each reach a specific photostationary state (pss) at long irradiation time (Figure 5). The initial concentration $C_{X_{II}}^{335}\left(0\right)$ was varied between $2.5\times 10^{-4}\;M$ and $6.04\times 10^{-6}\;M$, whereas $C_{X_{I}}^{335}\left(0\right)$ remained constant at $4.4\times 10^{-5}\;M$.

Each of the traces of the four reactants and products were fitted by the same Equation (7) (that were also used to fit their traces when obtained for single reactions, i.e., each individual reaction, not in a mixture). Hence, the overall pattern of each species’ kinetic trace was not affected by varying the initial concentration of one reactant of the mixture. The good fittings of the traces of the mixtures were indicated by similar quality of the assessing criteria ($r^{2},\;SSE$ and RMSE) to those characterizing the single reactions (see Supplementary Information, Section S3a).

**Figure 6:**
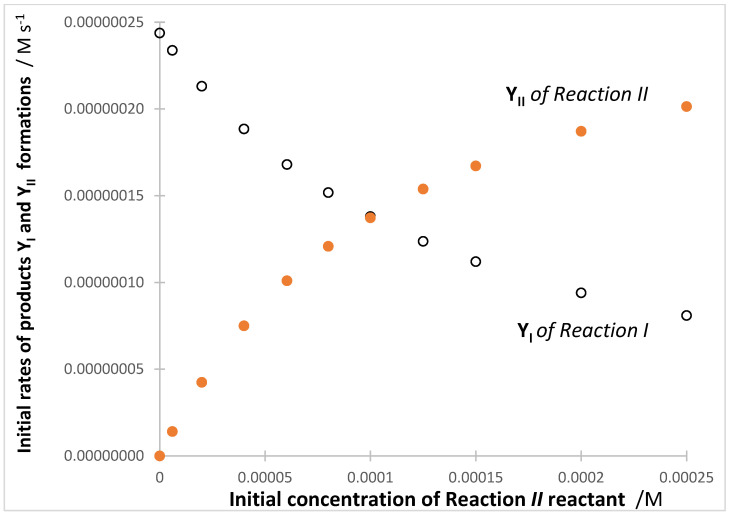
Evolution of the initial rate values of the formation of products $Y_{I}$ (circles) and $Y_{II}$ (dots) with an increasing initial concentration of $X_{II}$ ($C_{X_{II}}^{335}\left(0\right)$) while $C_{X_{I}}^{335}\left(0\right)=4.4\times 10^{-5}\;M$ is invariant. isolated reaction in the presence of SPMs (Figure 5). *Reaction II*, hence, seems to somewhat act as if it was an SPM for *Reaction I* in the mixture. The initial rates of the products have an opposite sign to those of the reactants, as $r_{0,X_{I}}^{335}=-\;r_{0,Y_{I}}^{335}$ and $r_{0,X_{II}}^{335}=-\;r_{0,Y_{II}}^{335}$.

In the absence of SPM, the variation of $C_{X_{II}}^{335}\left(0\right)$ induced a change in the rate of the reactions. Notably, the initial reactions’ rates of the binary components were variably affected. It is observed that the initial rate of the reactant of the second system, $r_{0,X_{II}}^{335}$, increased with increasing the concentration of that initial reactant ($C_{X,II}^{335,T}\left(0\right)$), whereas that of the first system, $r_{0,X_{I}}^{335}$ (whose constant $C_{X,I}^{335,T}\left(0\right)=4.4\times 10^{-5}\;M$), decreased in the same conditions (Figure 6).

This peculiar behavior is different from the one observed for SPMs effect, where both initial rates, $r_{0,X_{I}}^{335}$ and $r_{0,X_{II}}^{335}$, decreased (Figure 3). However, both $r_{0,X_{\chi }}$ behaviors can readily be interpreted by photokinetic considerations.

Indeed, let us first observe that the variation in the photokinetic factor of the binary mixture, $PKF^{335}\left(0\right)$ (Equation (2)), decreases with increasing $C_{X_{II}}^{335}\left(0\right)$. A decrease of $PKF^{335}\left(0\right)$ means that less light is absorbed by each species of the mixture at the irradiation wavelength ($\lambda_{irr}=335\;nm$), and hence reactivity should, in principle, reduce.

This is effectively the case of *Reaction I*, whose initial reactant-rate dependence on $C_{X_{II}}^{335}\left(0\right)$, is exclusively expressed in the formula of $PKF^{335,T,2}\left(0\right)$ (Equation (2)). The initial rate value ($r_{0,X_{I}}^{335}$) evolves from its non-zero and negative initial value (when $C_{X_{II}}^{335}\left(0\right)=0$ and $C_{X_{I}}^{335}\left(0\right)\neq 0$) to approaching a zero value ($r_{0,X_{I}}^{335}\to -0$) for high values of $C_{X_{II}}^{335}\left(0\right)$. Concomitantly, the positive values of the initial rate of its product, $r_{0,Y_{I}}^{335}=-\;r_{0,X_{I}}^{335}$, undergoes a smooth decrease, towards the zero value, in the same conditions (Figure 6). Hence, the higher $C_{X_{II}}^{335}\left(0\right)$ the lower the initial reactant-rate of the other reaction, i.e., $-\;r_{0,X_{I}}^{335}$.

The assessment of the photoreactivity of *Reaction I* might be conducted on analyzing the rate of the reactant as expressed in Equation (4), considered at a given reaction time ($t_{\alpha }$) when $C_{X_{II}}^{335}\left(0\right)$ increases. It is then obvious that $r_{X_{I}}^{335}(t_{\alpha })$ varies as the product involving the photokinetic factor of the mixture, $PKF^{335,T,2}\left(t_{\alpha }\right)$ and a factor dependent on $C_{X_{I}}^{335}\left(t_{\alpha }\right)$ (see Supplementary Information, Section S3b). Both these terms’ values should reduce when $C_{X_{II}}^{335}\left(0\right)$ increases (proportionally to $r_{0,X_{I}}^{335}$), and therefore, causing a slowdown of *Reaction I* reactivity (see Supplementary Information, Figure S2).

For this *Reaction I* in a mixture, both its initial reactant consumption rate and reaction rate decrease with increasing $C_{X_{II}}^{335}\left(0\right)$ ($C_{X_{I}}^{335}\left(0\right)$ being constant). This typical behavior is the same as that of an isolated reaction in the presence of *SPM*s (Figure 5). *Reaction II*, hence, seems to somewhat act as if it was an SPM for *Reaction I* in the mixture. Such observation is corroborated by experimental data for both the photodegradation of polychlorinated naphthalenes in mixtures [78], the sunscreen butyl methoxy-dibenzoyl methane in the presence of increasing concentration of bis-ethylhexylphenol methoxyphenyl triazine [37], UV/TiO_2_ photodegradation of binary and ternary mixtures involving metronidazole, ciprofloxacin, and/or sulfamethoxazole [41], and the depression in photodegradation of phenol, o-cresol, and 4-chlorophenol in binary mixtures [79].

The behavior of *Reaction II* in the mixture is slightly different to that of *Reaction I*. Here, the initial reactant consumption rate ($-\;r_{0,X_{II}}^{335}$) increases with $C_{X_{II}}^{335}\left(0\right)$. Photokinetically, this is explained by the fact that $r_{0,X_{II}}^{335}$ vary as the product, $C_{X_{II}}^{335}\left(0\right)\times PKF^{335,T,2}\left(0\right)$, according to the initial reactant-rate equation (Equation (5)). Despite the reduction recorded on the mixture photokinetic factor ($PKF^{335,T,2}$), overall, the latter product value increases with increasing $C_{X_{II}}^{335}\left(0\right)$ (see Supplementary Information, Figure S5). In this case, $r_{0,X_{II}}^{335}$ has an initial value of zero when $C_{X_{II}}^{335}\left(0\right)=0$, but approaches a negative non-zero limit-value, $r_{0,X_{II}}^{335}\to -\;\Phi_{X_{II}\;⟶\;Y_{II}}^{335}\;A_{X_{II}}^{335}(0)/A_{tot}^{335}(0)$, when $C_{X_{II}}^{335}\left(0\right)\to +\infty$ (and $r_{0,Y_{II}}^{335}$ smoothly increases (Figure 6)). Such behavior was observed for single reactions, such as for the first-order degradation of Metronidazole, whose initial rate increased with increasing its initial concentration, both when directly photolyzed and photo-catalytically degraded under different conditions and catalysts [71]. A similar initial rate increase was reported for both solophenyl green direct and catalytic photodecolorization [80].

**Figure 7:**
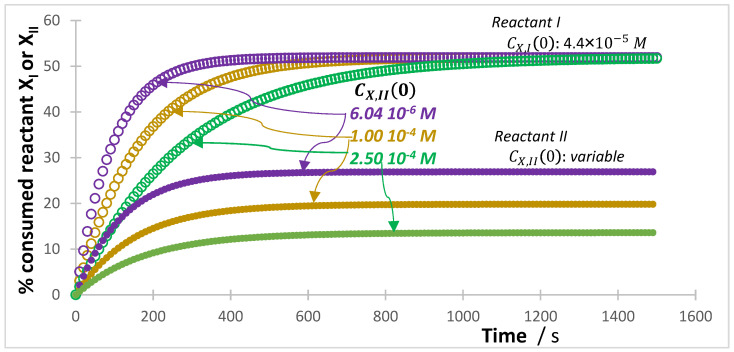
Evolution of the % consumed reactants of *Reaction I* and *II* with time. Three initial concentrations of reactant of *Reaction II* are shown ($C_{X,I}\left(0\right)\;\mathrm{is}\;\mathrm{constant}\;\mathrm{at}\;4.4\times 10^{-5}\;M$).

In terms of reactivity, it was previously shown that by increasing the reactant’s initial concentration of single reactions, the photoreactivity of photo and photothermal reactions undergo photostabilization [53,54,55,64,77]. Direct and photo-catalyzed degradation of metronidazole showed a clear reduction in reactivity for higher values of initial concentration [71]. The photocatalytic removal efficiency of sulfamethoxazole decreased with increased initial concentration [41]. Even though this conclusion was drawn for isolated reactions, it is expected to be still valid for mixtures, where the presence of other mixture components will accentuate such reduction in reactivity in proportion to the fraction of light they absorb. The best way to visualize such a trend is, perhaps, achieved by plotting the percentage of the reactant ($X_{I}$ and/or $X_{II}$) consumed over time for the mixture reactions, with this quantity (${\%}_{consd.}(X_{\chi })$) being calculated by, ${\%}_{consd.}(X_{\chi })=100\times (C_{X_{\chi }}^{335}(0)-C_{X_{\chi }}^{335}\left(t\right))/C_{X_{\chi }}^{335}\left(0\right)$. In this format, the direct comparison of kinetic traces is possible for reactions performed at very different initial concentrations (the concentration traces are difficult to compare quantitatively, see Figure S3). This strategy is useful in facilitating the plotting of the ${\%}_{consd.}(X_{\chi })\;$ for the reactants of the mixture’s components. In fact, data of several experiments can be visualized, as for instance, those recovered at three initial concentrations of the reactant of one of the mixture’s components, e.g., $C_{X_{II}}^{335}\left(0\right)$. Figure 7 gives the curves for the mixture depicted in Scheme 2.

As can be seen, the ultimate value of ${\%}_{react}(X_{I})$ for *Reaction I* (at t=∞), is the same irrespective of the initial concentration of $X_{II}$. This results from $C_{X_{I}}^{335}\left(0\right)$ being constant at $4.4\times 10^{-5}\;M$. However, the higher $C_{X_{II}}^{335}\left(0\right)$, the slower *Reaction I*, i.e., the slower the evolution of ${\%}_{consd.}\left(X_{I}\right)=f(t)$. A trend that corroborates the conclusion reached above, as *Reaction II* is an effective SPM to *Reaction I*.

Figure 7 also shows ${\%}_{consd.}(X_{II})$ for *Reaction II*. Here, the final value reached by the curve ${\%}_{consd.}\left(X_{II}\right)=f(t)$, recorded for each experiment, is different for the different values of $C_{X_{II}}^{335}\left(0\right)$. In fact, the final value reached by the curve of ${\%}_{consd.}\left(X_{II}\right)$ is inversely proportional to $C_{X_{II}}^{335}\left(0\right)$, meaning that more of the reactant is transformed when the $C_{X_{II}}^{335}\left(0\right)$ is smaller, and therefore, the reaction is faster (i.e., has a shorter half-life time, $t_{\frac{1}{2}}$). This confirms, on one hand, an autophotostabilization of *Reaction II* due to increasing $C_{X_{II}}^{335}\left(0\right)$, and on the other hand, an improved stabilization of this photoreaction by the presence of *Reaction I*, which in this case, plays the role of an SPM to *Reaction II*.

Experimental data relative to the effect of initial concentration on the kinetic features of each component of a mixture are very scarce in the literature. However, the results of some reported examples align with the above conclusions. For instance, a decline in the photodegradation reaction rate was observed for the binary mixture of 4-chlorophenol (whose initial concentration was constant, $C_{X_{I}}\left(0\right)=0.3\;mM$), in the presence of increasing concentration of phenol ($C_{X_{II}}\left(0\right)=0,\;0.3,\;0.45\;and\;0.9\;mM$). The pseudo-first-order rate-constants recorded for both 4-chlorophenol ($k_{I}=1.66,\;1.06,\;and\;0.34\;h^{-1}$, respectively) and phenol ($k_{II}=\;0.83,\;0.5,\;and\;0.27\;h^{-1}$, respectively) indicated between 70 and 80% decrease. A similar trend was also observed by the authors for the phenol/o-cresol mixture in the same condition of the latter mixture [79].

The kinetic phenomenon can also be rendered by considering the ratio of the initial rates ($r_{0,X_{II}}^{335}/r_{0,X_{I}}^{335}$), that must evolve as either $C_{X_{II}}^{335}\left(0\right)$ when $C_{X_{I}}^{335}\left(0\right)$ is constant or in general, as the ratio of the initial concentrations of the reactants (Equation (12), where the expression in brackets being always constant).

(12) $$\frac{Theo:\;r_{0,X_{II}}^{335}}{Theo:\;r_{0,X_{I}}^{335}}=\frac{-\Phi_{X_{II}\;⟶\;Y_{II}}^{335}\;P_{a_{Y_{0,II}}}^{\lambda_{irr}}\left(0\right)}{-\Phi_{X_{I}\;⟶\;Y_{I}}^{335}\;P_{a_{Y_{0,I}}}^{\lambda_{irr}}\left(0\right)}=\left(\frac{\Phi_{X_{II}\;⟶\;Y_{II}}^{335}\;\varepsilon_{X_{II}}^{335}}{\Phi_{X_{I}\;⟶\;Y_{I}}^{335}\;\varepsilon_{X_{I}}^{335}}\right)\;\frac{C_{X_{II}}^{335}\left(0\right)}{C_{X_{I}}^{335}\left(0\right)}\;$$

**Figure 8:**
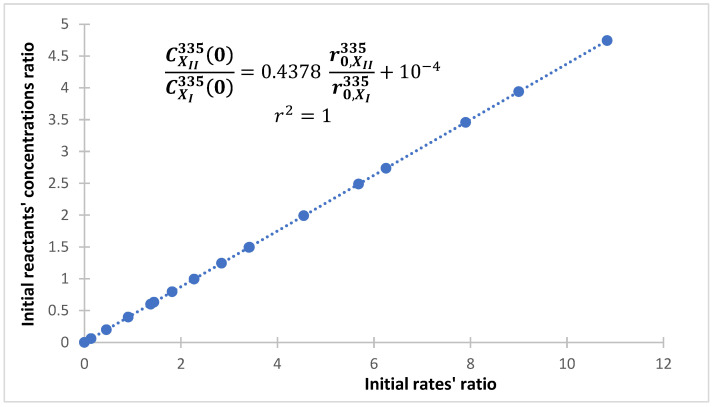
A linear relationship between the ratios of the initial rates and the initial concentration of the reactants of the reactions components, as predicted by Equation (12). A binary mixture of photoreversible reactions (Scheme 2) under monochromatic irradiation at 335 nm. The set of points include the case where is $C_{X_{I}}^{335}\left(0\right)$ is variable $0\;M>C_{X_{I}}^{335}\left(0\right)>25\times 10^{-5}\;M$ and $C_{X_{II}}^{335}\left(0\right)$ is constant ($2\times 10^{-5}\;M$)*,* including cases where both initial concentrations are different, $C_{X_{I}}^{335}\left(0\right)\neq C_{X_{II}}^{335}\left(0\right)$ as those shown in Table 1.

Figure 8 shows the linear relationship for the binary system depicted in Scheme 2 considered for various $C_{X_{I}}^{335}\left(0\right)$ and $C_{X_{II}}^{335}\left(0\right)$ values. Equation (12) stands for any values of the initial concentrations $C_{X_{I}}^{335}\left(0\right)$ and $C_{X_{II}}^{335}\left(0\right)$. The values of the ratios for several initial concentrations are shown in Table 1. The data of Table 1 fall on the line of Figure 8. This fact is important in the sense that it provides an easy way to check whether the reactions involved in the mixture are independent, by just plotting a few points.

In agreement with Equation (12), linear patterns are worked out from the experimental data reported for different ratios of phenol/o-cresol and phenol/4-chlorophenol binary mixtures [79] (see Supplementary Information, Section S3d and Figure S6).

For practical reasons, it is useful to consider the variation in the initial rate of the total absorbance trace (i.e., the trace that includes the absorbances of the medium species absorbing at the observation wavelength, $\lambda_{obs}$, with $\lambda_{obs}$ and $l_{obs}$ are not necessarily equal to $\lambda_{irr}$ and $l_{irr}$, respectively). For the case of the mixture of Scheme 2, Equation (6) gives

(13) $$\begin{array}{l} Theo:\;r_{0,A}^{\lambda_{obs},T,n_{mix}}=\\ l_{obs}\left(\varepsilon_{X_{I}}^{\lambda_{obs}}-\varepsilon_{Y_{I}}^{\lambda_{obs}}\right)\;Theo:\;r_{0,X_{I}}^{\lambda_{irr},T,n_{mix}}+l_{obs}\;\left(\varepsilon_{X_{II}}^{\lambda_{obs}}-\varepsilon_{Y_{II}}^{\lambda_{obs}}\right)\;Theo:\;r_{0,X_{II}}^{\lambda_{irr},T,n_{mix}} \end{array}$$

**Figure 9:**
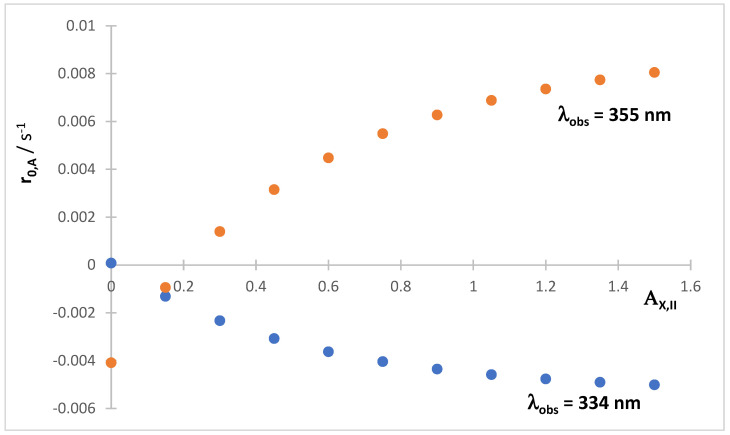
Examples of the variation in the initial rate measured at two different observation wavelengths, for the total absorbance traces of the mixture (shown in Scheme 2), with the initial absorbance of *Reaction II* reactant ($A_{X_{II}}\left(0\right)$).

The value of $r_{0,A}^{\lambda_{obs}}$ can be either, or both, positive or negative, when $C_{X_{I}}^{335}\left(0\right)$ is changed. The sign of $r_{0,A}^{\lambda_{obs}}$ is determined by both the signs of the differences in the absorption coefficients $\left(\varepsilon_{X_{\chi }}^{\lambda_{obs}}-\varepsilon_{Y_{\chi }}^{\lambda_{obs}}\right)$ at the observation wavelength and the relative magnitudes of the products $r_{0,X_{\chi }}^{\lambda_{irr}}\left(\varepsilon_{X_{\chi }}^{\lambda_{obs}}-\varepsilon_{Y_{\chi }}^{\lambda_{obs}}\right)$ (since $r_{0,X_{\chi }}^{\lambda_{irr}}$ have negative values). Figure 9 depicts the variations of $r_{0,A}^{\lambda_{obs}}$ measured for two $\lambda_{obs}$ (with $l_{obs}$ being the same).

Owing to the versatile variations in both $r_{0,X_{\chi }}^{\lambda_{irr}}$ (Figure 6) and $r_{0,A}^{\lambda_{obs}}$ (Figure 9), the acquisition of two values of these parameters (e.g., at $C_{X_{II}}^{335}\left(0\right)=0$ and another at $C_{X_{II}}^{335}\left(0\right)\neq 0$) might not facilitate interpretation. It is hence recommended to prospect a series of concentration values covering the largest possible concentration range.

It is interesting to notice that for our mixture, the values of absorbance traces $r_{0,A}^{\lambda_{obs}}$ obtained for different concentration $C_{X_{I}}\left(0\right)$ and at different observation wavelength, $\lambda_{obs}$, correlate linearly. Linear trends are also observed for, on one hand, the values of $r_{0,X_{I}}^{\lambda_{irr}}$ against $r_{0,X_{II}}^{\lambda_{irr}}$ measured on the concentration traces of the reactants, and on the other hand, the $r_{0,Y_{I}}^{\lambda_{irr}}$ against $r_{0,Y_{II}}^{\lambda_{irr}}$ measured on the concentration traces of the products.

The kinetic features reviewed in this section (e.g., decreasing rates of both reactions, as well as the opposed pattern of evolution for the initial reactants’ consumption rates), become the typical trends for a binary mixture when the initial concentration of one or both components reactants’ is varied.

This rationalization of the binary mixture photokinetics is consistent as long as the involved reactions are independent from each other. Whenever the patterns shown here are not observed, it is legitimate to consider the involvement of other processes. In such a case, the mixture reactions are dependent, e.g., by the occurrence of photo- or chemical reactions, or physical interactions between the species of the mixture components present in the medium. Such reactions might further be evidenced by the emergence of new reaction products in the medium of the mixture that were not previously observed for the individual reactions considered separately [2]. For instance, an acceleration of the decay of ethylhexyl methoxycinnamate in the presence of butyl methoxy-dibenzoyl methane was attributed to a [2+2]-cycloaddition [37], the sulfamethoxazole increased degradation in the presence of benzophenone was assigned to a triplet photosensitization by the latter component of the binary mixture [81], and in the case of dihydralazine binary mixture with hydrochlorothiazide, both components underwent faster photodegradation than each alone [3].

#### 2.7.2. A Ternary Mixture Under Polychromatic Light

**Scheme 3:**
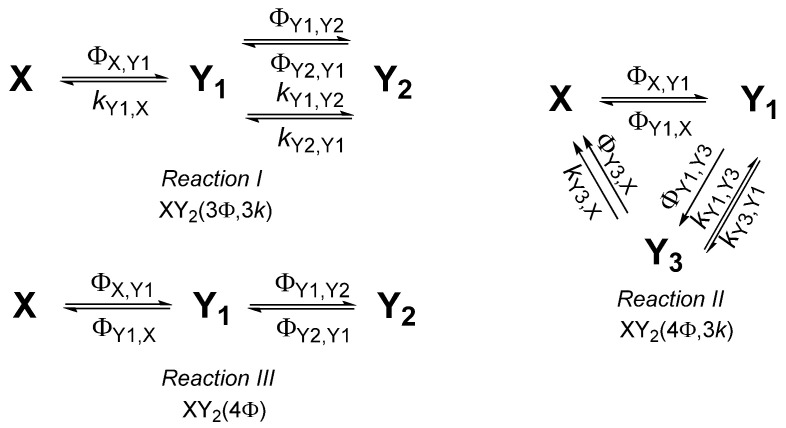
Components of the ternary mixture.

The ternary mixture used here for illustration encompasses three different photo- and photothermal reactions $XY_{2}(3\Phi ,3k)$, $XY_{2}\left(4\Phi ,3k\right)$ and $XY_{2}(4\Phi )$, each involving three reactive species (Scheme 3).

**Figure 10:**
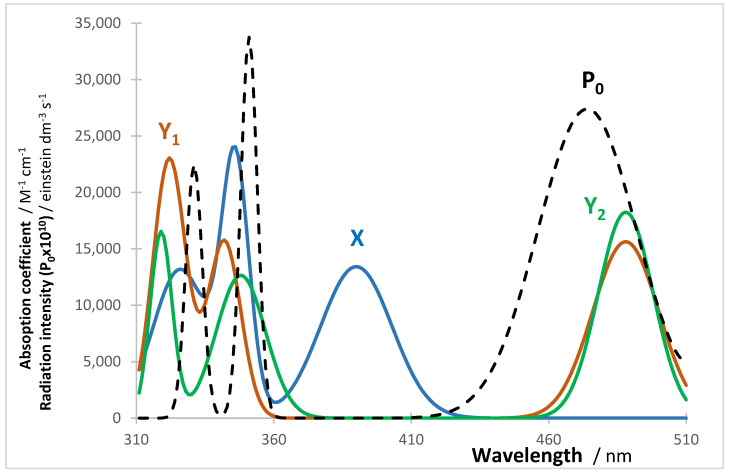
Absorption coefficients spectra and the lamp profile ($P_{0}$) for *Reaction I* (Scheme 3).

The thermally stable reactants of these reactions absorb in the short wavelength region (e.g., in the UV) whereas the photo and thermally active products absorb in the long wavelength region (e.g., in the Visible) (Figure 10). These characteristics are typical of photochromes [21,82,83].

**Figure 11:**
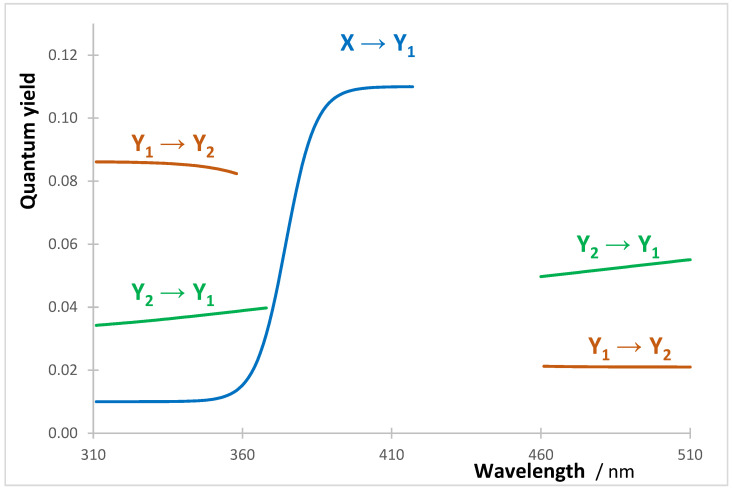
Quantum yield patterns for the three photoreactions of *Reaction I* (Scheme 3). The rate-constants of the thermal reaction-steps are $k_{Y_{1,I}\;⟶\;X_{I}}^{∆}=0.021\;s^{-1}$, $k_{Y_{1,I}\;⟶\;Y_{2,I}}^{∆}=0.0096\;s^{-1}$ and $k_{Y_{2,I}\;⟶\;Y_{1,I}}^{∆}=0.0057\;s^{-1}$.

The mixture is subjected to a polychromatic light that variably activates reactants and products (with $l_{irr}=0.81\;cm$). The wavelength-dependent quantum yields of the photoreaction-steps for each reaction follow different patterns and are different between reactions. The quantum yields of the three photoreaction-steps of *Reaction I* are presented in Figure 11, whereas those of the two other reactions together with the species spectra are available in Supplementary Information (Section S4).

The traces of the species concentrations, absorbances, and total absorbances were fitted by Equations (7) and (9) that encompass three mono-Φ-order and three first-order terms or less (i.e., for all cases, $i_{\Phi ,j,\chi }\leq n_{\Phi ,j,\chi }$ and $i_{∆,j,\chi }\leq n_{∆,j,\chi }$).

Irradiation with polychromatic light does not modify the overall Φ-order kinetic behavior that was observed for photoreaction-steps when the mixture is irradiated by monochromatic light. An aspect confirming a previously set conclusion stating that “*the* Φ*-order kinetic pattern of an individual reaction is not affected by the type of (non-isosbestic mono- or polychromatic) light used for irradiation*” [53,54,55,58]. This statement is extended, by the present results, to include photothermal reactions within a mixture (whose kinetics, hence, obey the combined Φ-/first-order patterns, which are analogous to those recorded for the individual reactions). This finding strongly indicates that the typical Φ-order kinetic behavior, of a given species, is preserved whether it is involved in a mixture or not. A reaction rate is, hence, only affected by the amount of light received by its species.

**Figure 12:**
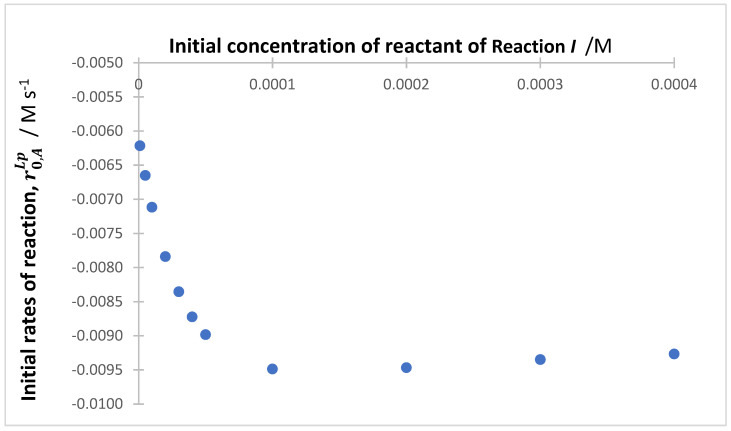
Evolution of the initial rates of the reaction, $r_{0,A}^{Lp}$, with an increasing initial concentration of $X_{I}$ ($C_{X_{I}}^{Lp}\left(0\right)$) while $C_{X_{II}}^{Lp}\left(0\right)=8.41\times 10^{-6}\;M$ and $C_{X_{III}}^{Lp}\left(0\right)=2.36\times 10^{-5}\;M$ is kept invariant.

The changes occurring on reactivity of the species in ternary mixtures, due to variation in a given reaction parameter, are similar to those observed for the binary ones. In the presence of SPMs, the reactions gradually slow down with increasing SPMs absorbance. Likewise, the initial consumption rate of the reactant whose initial concentration increased (in the absence of SPMs) is characterized by an increase whereas those of the two other reactions decrease (see Supplementary Information, Figure S12). Linear relationships characterize the plots of the ratios of the initial concentrations ($C_{X_{I}}^{335}\left(0\right)/C_{X_{II}}^{335}\left(0\right)$ or $C_{X_{I}}^{335}\left(0\right)/C_{X_{III}}^{335}\left(0\right)$) against ratios of the initial rates ($r_{0,X_{I}}^{Lp}/r_{0,X_{II}}^{Lp}$ and $r_{0,X_{I}}^{Lp}/r_{0,X_{III}}^{Lp}$, respectively) (see Supplementary Information, Figure S13, Equations (S24) and (S25)). The variation in the absolute value of the initial reaction rate, obtained from the total absorbance trace ($-\;r_{0,A}^{Lp}$) for the mixture in Scheme 3, with an increasing initial concentration of $X_{I}$ (while the initial concentrations of the two other reactants are invariable), is characterized by a steep increase up to $C_{X_{I}}^{335}\left(0\right)\approx 10^{-4}\;M$, followed by a slow decrease for higher concentrations of $X_{I}$ (Figure 12). Such a non-monotonical pattern of $r_{0,A}^{Lp}=f(C_{X_{I}}^{335}\left(0\right))$ is fully explained photokinetically and should not be misinterpreted as due to other or new processes occurring in the mixture medium.

## 3. Experimentals

The combination or mixture of photothermal reactive systems envisaged in the present study are either binary or ternary mixtures ($n_{mix}=2\;or\;3$) where, respectively, two or three independent photo- and/or photothermal reactions are concomitantly present in a reactor. The reactive systems studied within a given reactor are each identified by the label χ which varies between *I* and *III* (I≤χ≤III) depending on the number of components $n_{mix}$ in the mixture. Each reactive system (χ) in the mixture involves $n_{sp,\chi }$ species (i.e., its reactant and all its products labeled as $Y_{j,\chi }$ with $0\leq j\leq n_{sp,\chi }$ and where the reactant is $Y_{0,\chi }=X_{\chi }$). The reaction species are labeled according to the convention previously adopted for the Φ-shaped mechanism [53]. The reactor might contain, at any time t, up to $n_{sp,tot}=n_{sp,I}+n_{sp,II}+n_{sp,III}$ coexisting species. Reactants, products, and their features are designated relative to the reactive system they belong to, such as $Y_{j,\chi }$, $\varepsilon_{Y_{j,\chi }}^{\lambda_{obs}}$, $\Phi_{Y_{j,\chi }\;⟶\;Y_{j^{\prime },\chi }}^{\lambda_{irr}}$…, etc.

The reactor is slab-shaped, vigorously stirred, and continuously irradiated. The irradiation light can either be monochromatic or polychromatic and is supposed to be collimated. The parameters of the reactions except for the thermal rate-constants (e.g., $k_{Y_{j^{\prime },\chi }\;⟶\;Y_{j,\chi }}^{∆}$) are all considered to be wavelength-dependent (including the quantum yields of the photochemical reaction-steps).

The system of rate-equations describing the kinetics is built on the principles adopted in our previous work [53,54,55], using the same labeling system with the addition of the specific-component label (χ) for each system in the mixture, and a mathematical amendment of the equations that relays the multiple/simultaneous absorption of the light by the species present in the reactor. Each reactor is described by a system of rate-equations, equal to the sum of the subsystems of rate-equations of each individual reactive system, i.e., $n_{sp,tot}$ equations.

The kinetic traces of each individual species in the reactor under investigation were numerically generated by the fourth-order Runge–Kutta (RK4) numerical integration method. The RK4 calculations were performed by a homemade program running on the Microsoft Excel VBA platform.

The numerical integration is used here both as a tool for modeling and testing. RK4-calculated traces that are a reference for testing the performance of the explicit integrated rate-law formula (vide infra Equation (7)). The latter is proposed to describe the species’ concentration traces.

Each species in the reactor is characterized by an explicit formula, Equation (7), that involves $i_{\Phi ,j,\chi }$ mono-Φ-order terms (of the form, $w\;Log\left(1+cc\;e^{-k\;t}\right)$), and $i_{∆,j,\chi }$ mono-exponential terms (of the form, $w\;e^{-k\;t}$). The former number of terms ($i_{\Phi ,j,\chi }$), corresponds exclusively to the photochemical reaction-steps starting or ending at the considered species $Y_{j,\chi }$, $n_{\Phi ,j,\chi }$. This number cannot exceed the total number of photoreaction-steps in the mechanism of the considered reaction χ, $n_{\Phi ,\chi }$, but it can be lower than $n_{\Phi ,\chi }$ or even $n_{\Phi ,j,\chi }$, depending on the reactive medium. Similarly, the latter, $i_{∆,j,\chi }$, corresponds to the number of thermal reaction-steps ($n_{∆,j,\chi }$) starting or ending at the considered species $Y_{j,\chi }$. It is most equal to the total number of thermal reaction-steps in the mechanism of the reaction ($n_{∆,\chi }$), but as $n_{\Phi ,j,\chi }$, can be less than both $n_{∆,\chi }$ and $n_{∆,j,\chi }$.

The fitting of each Equation (7) to the corresponding RK-generated traces (for the reactant $X_{\chi }=Y_{0,\chi }$ and each product $Y_{j,\chi }$ of a given reactive system) was performed by the curve fitting tool of an R2020b MatLab software based on a Levenberg–Marquardt Algorithm (LMA). The goodness of fit of the traces was evaluated by the squared correlation coefficients (*r*^2^), the sum of squares error (SSE), and the root-mean-square deviation (RMSD). The fitting parameters (w, cc and k) might be different for different traces (even for the same reaction).

The RK-calculations assume, de facto, that the concentrations of reactants and products present in the reactor belong to the respective linearity ranges of those species’ calibration graphs throughout the duration of the reaction (irrespective of the reaction investigated).

## 4. Conclusions

The photokinetics of mixtures was proven to be well described by the explicit formula (Equation (7)), and the quantification was hence facilitated. These tools, unprecedented in the literature, allow a better investigation of mixtures under either mono- or polychromatic irradiation, irrespective of the mechanisms governing the photothermal reactions involved in the mixture, regardless of whether the quantum yields of the photoreaction-steps are wavelength-dependent, and in the presence or the absence of spectator molecules. As such, they cover a large extent of conditions relevant for research and industry.

The strategy of using Runge–Kutta numerical integration to test explicit equations has proven to be of great efficacy. The combination of simulated data, the consistent photokinetic theoretical background, and the flexibility of the explicit equation have offered a full description of the photokinetics of mixtures.

The description is amended with quantifying tools that are useful for experimental investigation. The standard behavior of a mixture involving independent reactions is illustrated by means of typical patterns of the initial reactants’ consumption rates as shown in Figure 3 and Figure 6, and Figure S13, and by the verification of Equation (12) (and Equation (S25)). The retardation of the reactions can be evidenced by the reduction in the percentage consumptions of the individual components’ reactants, at a given reaction time, from the curves of the characteristic Figure 7.

As a general observation, the reactivities of the individual components in the mixture are slower than those of these components studied separately. The reduction in the reactivity is accentuated by increasing the initial concentrations of one or more of the mixture components.

From a practical point of view, the recommendation to boost the degradation efficiency of the mixture’s components is to consider lowering their concentrations and/or increasing the incident light intensity (as long as the medium temperature is maintained at a constant value). Such efficiency is also intimately dependent on the intrinsic features of the components within OSIA. The differences between the absorptivities and quantum yield values of the individual components of a mixture define their overall kinetic behavior and degradation. It is then important to characterize the individual components of a mixture separately, unraveling their absorption spectra, the values of the quantum yields of each photochemical reaction-step, and these quantum yields patterns with wavelength (e.g., by using the elucidation method and photokinetic tools previously established [55,58]).

Once such data is available, optimization of the behavior of the mixture for the desired application can be achieved through the modeling of the reaction by numerical methods—a strategy that should save both time and resources.

The results laid out here are thought to contribute to rationalizing photokinetics along the same concepts imposed in the sister field of thermal kinetics.

While the methods and concepts presented here fill a gap in the knowledge, photokinetics will benefit from further research on aspects not covered in the present work, including, for instance, assigning explicit equations to the coefficients of Equation (7), considering mixtures with interacting components, modeling mixture reactivity when taking into account the 3-D geometry of the sample holder, and/or the spatial distribution of the incident light impinging on the sample.

## Figures and Tables

**Table 1:** Examples of initial rates ratios for different initial concentrations. The binary system (Scheme 2) which has the same reaction conditions described in the text.

Case → Parameters ↓	1	2	3	4	5	6
$C_{X_{I}}^{335}\left(0\right)\;/M$	1.2 × 10^−5^	3 × 10^−5^	2.8 × 10^−5^	6.5 × 10^−5^	7 × 10^−5^	4.4 × 10^−5^
$C_{X_{II}}^{335}\left(0\right)\;/M$	1.3 × 10^−4^	2.7 × 10^−4^	2.21 × 10^−4^	2.22 × 10^−4^	1.01 × 10^−4^	6.04 × 10^−6^
$\frac{C_{X_{II}}^{335}\left(0\right)}{C_{X_{I}}^{335}\left(0\right)}$	10.83	9	7.9	3.42	1.44	0.137
$\frac{r_{0,X_{II}}^{335}}{r_{0,X_{I}}^{335}}$	4.743	3.941	3.456	1.495	0.631	0.0601

↓ reads the column and “→” reads the line.

## Data Availability

The original contributions presented in this study are included in the article. Further inquiries can be directed to the corresponding author.

## Supplementary Materials

The following supporting information can be downloaded at: https://www.mdpi.com/article/10.3390/molecules30204122/s1.
