# Supplementary material for: Photokinetics of Mixtures of Independent Photoreactions
Source: Molecules. 2025 Oct 17;30(20):4122. doi: 10.3390/molecules30204122 (PMC12565790; doi:10.3390/molecules30204122)
Supplement: Supplementary file 1 [file molecules-30-04122-s001.zip › molecules-3897124-supplementary.pdf]

Supplementary Information (SI)

Photokinetics of mixtures

Mounir MAAFI

Leicester School of Pharmacy, De Montfort University, The Gateway, Leicester, LE1 9BH, UK

Supplementary Information (SI)

Section S1. The binary $XY_7(14\Phi)$ system

The system involves two reactions obeying the same mechanism (*Scheme 1*) but are characterised by different properties as given in table S1. $P_0^{\lambda_{irr}}$ =3.33 10^{-6} einstein dm^{-3} s^{-1}, l_{irr} = 1 cm

Table S1.

Reaction I $(\chi = I)$									
	$C_{X_\chi}(0)$	$\varepsilon_{Y_{0,\chi}}^{\lambda_{irr}}$	$\varepsilon_{Y_{1,\chi}}^{\lambda_{irr}}$	$\varepsilon_{Y_{2,\chi}}^{\lambda_{irr}}$	$\varepsilon_{Y_{3,\chi}}^{\lambda_{irr}}$	$\varepsilon_{Y_{4,\chi}}^{\lambda_{irr}}$	$\varepsilon_{Y_{5,\chi}}^{\lambda_{irr}}$	$\varepsilon_{Y_{6,\chi}}^{\lambda_{irr}}$	$\varepsilon_{Y_{7,\chi}}^{\lambda_{irr}}$
	9.41 10^{-6}	14321	20012	20631	17439	17439	20926	16508	21345
	$\Phi_{X\to Y_1}^{\lambda_{irr}}$	$\Phi_{Y_1\to X}^{\lambda_{irr}}$	$\Phi_{X\to Y_3}^{\lambda_{irr}}$	$\Phi_{Y_3\to X}^{\lambda_{irr}}$	$\Phi_{Y_1\to Y_2}^{\lambda_{irr}}$	$\Phi_{Y_2\to Y_1}^{\lambda_{irr}}$	$\Phi_{Y_1\to Y_3}^{\lambda_{irr}}$	$\Phi_{Y_3\to Y_1}^{\lambda_{irr}}$	
	0.44	0.67	0.23	0.71	0.42	0.66	0.56	0.18	
	$\Phi_{Y_1\to Y_4}^{\lambda_{irr}}$	$\Phi_{Y_1\to Y_5}^{\lambda_{irr}}$	$\Phi_{Y_2\to Y_3}^{\lambda_{irr}}$	$\Phi_{Y_3\to Y_2}^{\lambda_{irr}}$	$\Phi_{Y_3\to Y_6}^{\lambda_{irr}}$	$\Phi_{Y_3\to Y_7}^{\lambda_{irr}}$			
	0.27	0.7	0.35	0.77	0.87	0.59			
Reaction II $(\chi = II)$									
	$C_{X_\chi}(0)$	$\varepsilon_{Y_{0,\chi}}^{\lambda_{irr}}$	$\varepsilon_{Y_{1,\chi}}^{\lambda_{irr}}$	$\varepsilon_{Y_{2,\chi}}^{\lambda_{irr}}$	$\varepsilon_{Y_{3,\chi}}^{\lambda_{irr}}$	$\varepsilon_{Y_{4,\chi}}^{\lambda_{irr}}$	$\varepsilon_{Y_{5,\chi}}^{\lambda_{irr}}$	$\varepsilon_{Y_{6,\chi}}^{\lambda_{irr}}$	$\varepsilon_{Y_{7,\chi}}^{\lambda_{irr}}$
	8.2 10^{-6}	22122	20987	17654	12345	9876	6754	13244	7658
	$\Phi_{X\to Y_1}^{\lambda_{irr}}$	$\Phi_{Y_1\to X}^{\lambda_{irr}}$	$\Phi_{X\to Y_3}^{\lambda_{irr}}$	$\Phi_{Y_3\to X}^{\lambda_{irr}}$	$\Phi_{Y_1\to Y_2}^{\lambda_{irr}}$	$\Phi_{Y_2\to Y_1}^{\lambda_{irr}}$	$\Phi_{Y_1\to Y_3}^{\lambda_{irr}}$	$\Phi_{Y_3\to Y_1}^{\lambda_{irr}}$	
	0.33	0.28	0.56	0.45	0.89	0.42	0.34	0.61	
	$\Phi_{Y_1\to Y_4}^{\lambda_{irr}}$	$\Phi_{Y_1\to Y_5}^{\lambda_{irr}}$	$\Phi_{Y_2\to Y_3}^{\lambda_{irr}}$	$\Phi_{Y_3\to Y_2}^{\lambda_{irr}}$	$\Phi_{Y_3\to Y_6}^{\lambda_{irr}}$	$\Phi_{Y_3\to Y_7}^{\lambda_{irr}}$			
	0.41	0.15	0.23	0.65	0.23	0.54			

The explicit equation used for the fitting, has the following form

$$C_{Y_{j,\chi}}^{\lambda_{irr},T,2}(t) = \omega_{j,\chi}^{\infty} + \omega_{1j,\chi}^{\Phi}\, Log\left(1 + cc_{j,\chi}^{\Phi}\, e^{-\,k_{1j,\chi}^{\Phi}\, t}\right) + \omega_{2j,\chi}^{\Phi}\, Log\left(1 + cc_{j,\chi}^{\Phi}\, e^{-\,k_{2j,\chi}^{\Phi}\, t}\right) + \omega_{3j,\chi}^{\Phi}\, Log\left(1 + cc_{j,\chi}^{\Phi}\, e^{-\,k_{3j,\chi}^{\Phi}\, t}\right) \qquad (\mathbf{S1})$$

The traces of *Reaction I* were presented on Fig.1 of the main text. The fitting parameters of the eight species traces are given hereafter in Table S2.

Table S2.

	$\omega_{j,\chi}^{\infty}$ $\times 10^6$	$cc_{j,\chi}^{\Phi}$	$\omega_{1j,\chi}^{\Phi}$	$k_{1j,\chi}^{\Phi}$	$\omega_{2j,\chi}^{\Phi}$	$k_{2j,\chi}^{\Phi}$	$\omega_{3j,\chi}^{\Phi}$	$k_{3j,\chi}^{\Phi}$	r^2	SSE $\times 10^{14}$	$RMSE$ $\times 10^9$
	Reaction I $(\chi = I)$										
$C_{Y_{0,\chi}}^{\lambda_{irr},T,2}(t)$	-	0.001077	0.01618	0.02883	0.003837	0.1245	-	-	1	3.174	5.639
$C_{Y_{1,\chi}}^{\lambda_{irr},T,2}(t)$	-	0.000374	0.00935	0.02883	-0.00534	0.1245	-0.0041	0.4372	0.999	1.590	3.999
$C_{Y_{2,\chi}}^{\lambda_{irr},T,2}(t)$	-	1.283	3.45e-6	0.02883	-4.397e-6	0.1245	9.499e-7	0.4372	0.999	94.23	3.074
$C_{Y_{3,\chi}}^{\lambda_{irr},T,2}(t)$	-	0.3789	6.99e-6	0.02883	-3.375e-6	0.1245	-4.02e-6	0.4372	1	208	45.68
$C_{Y_{4,\chi}}^{\lambda_{irr},T,2}(t)$	1.372	-3.623e-6	1.071	0.02883	-0.1986	0.1245	0.00792	0.4372	0.999	1.166	3.421
$C_{Y_{5,\chi}}^{\lambda_{irr},T,2}(t)$	3.563	0.2478	-4.25e-5	0.02883	3.62e-06	0.1245	1.902e-6	0.4372	1	1259	11.24
$C_{Y_{6,\chi}}^{\lambda_{irr},T,2}(t)$	2.662	0.06689	-1.17e-4	0.02883	2.167e-05	0.1245	1.893e-7	0.4372	1	27.94	1.671
$C_{Y_{7,\chi}}^{\lambda_{irr},T,2}(t)$	1.805	0.01134	-4.64e-4	0.02883	9.914e-05	0.1245	-7.83e-6	0.4372	1	809.4	90.15

The traces of *Reaction II* are shown on Figure S1 in the main text. The fitting parameters of the eight species traces are given in Table S3.

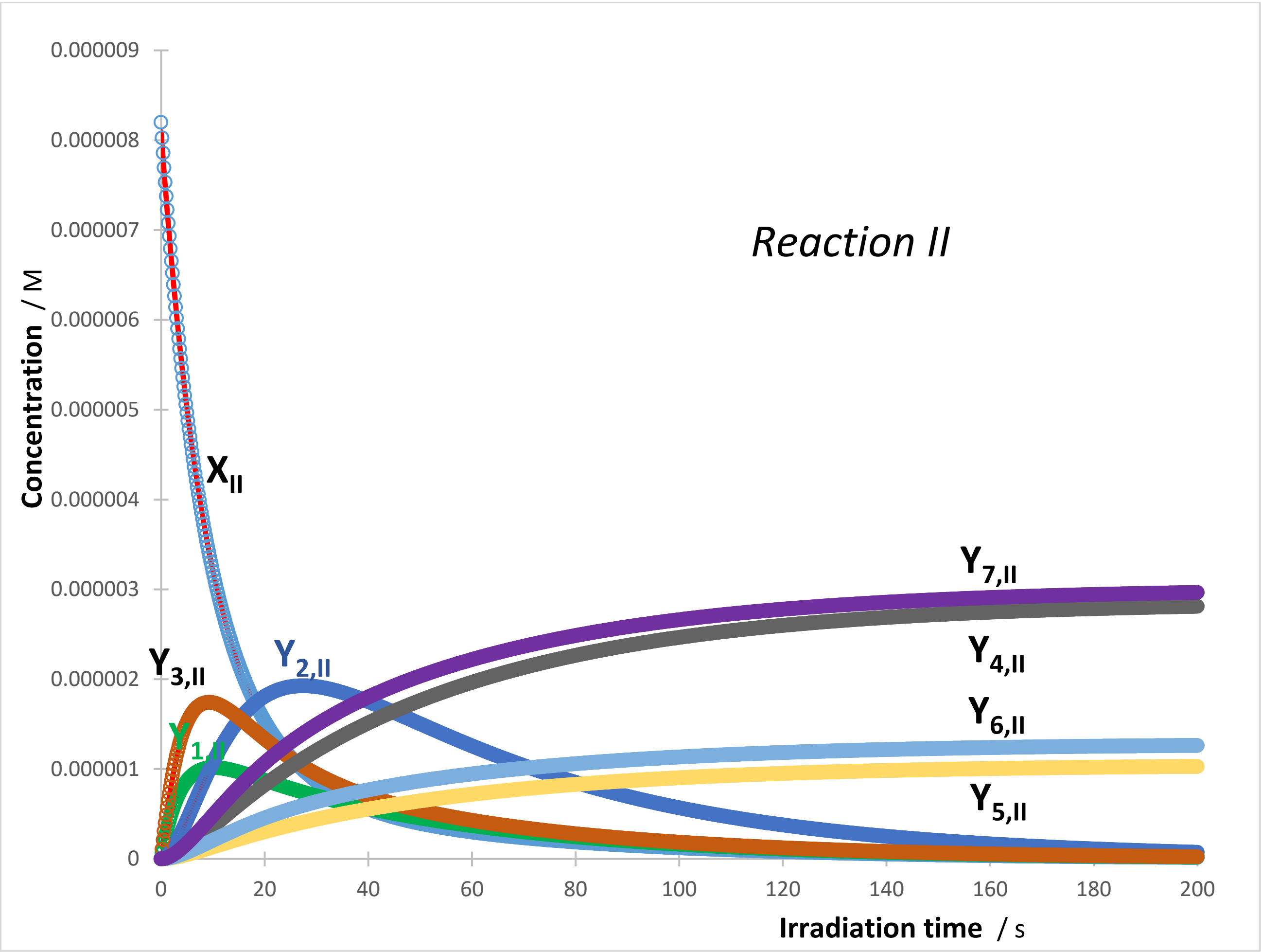

Figure S1

Table S3.

	$\omega_{j,\chi}^{\infty} \times 10^6$	$cc_{j,\chi}^{\Phi}$	$\omega_{1j,\chi}^{\Phi}$	$k_{1j,\chi}^{\Phi}$	$\omega_{2j,\chi}^{\Phi}$	$k_{2j,\chi}^{\Phi}$	$\omega_{3j,\chi}^{\Phi}$	$k_{3j,\chi}^{\Phi}$	r^2	$SSE \times 10^{14}$	$RMSE \times 10^9$
	Reaction I $(\chi = I)$										
$C_{Y_{0,\chi}}^{\lambda_{irr},T,2}(t)$	-	1.27E-5	0.2342	0.02397	1.237	0.1131			.9999	18.99	13.81
$C_{Y_{1,\chi}}^{\lambda_{irr},T,2}(t)$	-	5.70E-06	-0.444	0.2843	-0.1073	0.2844	0.5413	0.0213	.9997	2.803	5.31
$C_{Y_{2,\chi}}^{\lambda_{irr},T,2}(t)$	-	0.1796	1.96E-5	0.2843	-8.12E-05	0.08358	6.16E-05	0.0213	.9999	3.972	6.315
$C_{Y_{3,\chi}}^{\lambda_{irr},T,2}(t)$	-	1.976	-5.54E-6	0.3388	3.60E-06	0.0729	2.07E-06	0.0217	.9999	2.508	5.023
$C_{Y_{4,\chi}}^{\lambda_{irr},T,2}(t)$	2.86E-6	2.86E-06	2.86E-6	2.86E-6	2.86E-06	2.86E-6	2.86E-06	2.86E-6	1	0.03325	0.578
$C_{Y_{5,\chi}}^{\lambda_{irr},T,2}(t)$	1.05E-6	0.07532	1.75E-5	0.1413	-1.63E-05	0.1245	-3.48E-5	0.0205	1	0.001715	0.1314
$C_{Y_{6,\chi}}^{\lambda_{irr},T,2}(t)$	1.28E-6	0.02769	4.36E-5	0.2125	-5.07E-05	0.1245	-9.98E-5	0.0212	1	0.2869	1.699
$C_{Y_{6,\chi}}^{\lambda_{irr},T,2}(t)$	2.98E-6	-6.48E-06	0.1423	0.06229	-0.05888	0.1245	1.02	0.0226	.9998	9.937	9.998

Section S2. SPM effect on photoreactivity

The presence of increasing concentration of SPM in medium causes the total absorbance to increase, while the initial concentrations of the mixture components remain the same. The initial reactant rate is then written for a monochromatic irradiation, as

$$r_{0,X,\chi}^{\lambda_{irr},T,\chi} = -\,\Phi_{X_\chi \to Y_\chi}^{\lambda_{irr}}\, P_0^{\lambda_{irr}}\, \varepsilon_{X,\chi}^{\lambda_{irr}}\, l_{irr}\, C_{X_\chi}^{\lambda_{irr},T,2}(0)\, PKF^{\lambda_{irr},SPM}(0) \qquad (\mathbf{S2})$$

So, the value $-\,r_{0,X,\chi}^{\lambda_{irr},T,\chi}$ will decrease for higher values of SPM concentration, as does $PKF^{\lambda_{irr},SPM}(0)$ (since $C_{X_\chi}^{\lambda_{irr},T,2}(0)$ and the other coefficients of the equation are constant). This means that all the components of the mixture will see their $-\,r_{0,X,\chi}^{\lambda_{irr},T,\chi}$ decrease.

Each component of the mixture is deprived of a fraction of the incident light due to the presence of SPM. Therefore, each component reactivity will decrease (smaller rate-constants), and thus, longer half-life times for each component.

Section S3. The binary system under monochromatic light (*Scheme* 2 in the main text)

Section 3a. *Fitting data*

The explicit equations used for the fitting of reactant and product of Reaction I, has a single Φ-order term.

$$C_{Y_{j,\chi}}^{\lambda_{irr},T,2}(t) = \omega_{j,\chi}^{\infty} + \omega_{ij,\chi}^{\Phi}\, Log\left(1 + cc_{j,\chi}^{\Phi}\, e^{-\,k_{ij,\chi}^{\Phi}\, t}\right) \qquad (\mathbf{S3})$$

Whereas that of Reaction II, has one Φ-order and one mono-exponential terms.

$$C_{Y_{j,\chi}}^{\lambda_{irr},T,2}(t) = \omega_{j,\chi}^{\infty} + \omega_{ij,\chi}^{\Phi}\, Log\left(1 + cc_{j,\chi}^{\Phi}\, e^{-\,k_{ij,\chi}^{\Phi}\, t}\right) + \omega_{ij,\chi}^{\Delta}\, e^{-\,k_{ij,\chi}^{\Delta}\, t} \qquad (\boldsymbol{S4})$$

Fitting of the traces of the reactants of *reactions I and II* ($C_{Y_{0,I}}^{\lambda_{irr},T,2}(t)$ and $C_{Y_{0,II}}^{\lambda_{irr},T,2}(t)$) when the initial concentration of the reactant of *Reaction II* ($C_{Y_{0,II}}^{\lambda_{irr},T,2}(0)$) varies (while $C_{Y_{0,I}}^{\lambda_{irr},T,2}(0) = 4.4\ 10^{-5}\ M$, is constant), where P_0^{335} = 2.12 10^{-6} einstein $dm^{-3}\ s^{-1}$, l_{irr} = 1.58 cm, and $\lambda_{irr} = 335\ nm$.

Table S4.

$C_{Y_{0,II}}^{\lambda_{irr},T,2}(t) \times 10^5\ M$	0	0.604	2	4	6.04	8	10	12.5	15	20	25
Reaction I ($\chi = I$)											
$\omega_{j,\chi}^{\infty} \times 10^6$	0.2117	21.16	21.17	21.16	21.15	21.15	21.17	21.15	21.15	21.15	21.15
$cc_{j,\chi}^{\Phi}$	0.2098	0.02083	0.1818	0.00755	0.000905	0.00195	0.109	0.0011	0.01774	0.00693	0.0015
$\omega_{1j,\chi}^{\Phi} \times 10^4$	2.761	25.69	3.147	70.39	584.6	270.8	5.083	463.6	29.99	76.4	359.6
$k_{1j,\chi}^{\Phi}$	0.01172	0.01077	0.01013	0.00858	0.0076	0.00684	0.00636	0.0055	0.00502	0.00418	0.0036

r^2	1	1	1	1	1	1	1	1	1	1	1
SSE	8.507 10^{-19}	1.08 10^{-11}	2.801 10^{-17}	9.389 10^{-14}	8.688 10^{-14}	7.229 10^{-14}	2.277 10^{-18}	5.014 10^{-14}	2.546 10^{-14}	2.214 10^{-14}	1.755 10^{-14}
$RMSE$	7.607 10^{-11}	8 10^{-8}	4.336 10^{-10}	2.527 10^{-8}	2.431 10^{-8}	2.218 10^{-8}	1.245 10^{-10}	1.847 10^{-8}	1.316 10^{-8}	1.227 10^{-8}	1.093 10^{-8}
Reaction II $(\chi = II)$											
$\omega_{j,\chi}^{\infty} \times 10^6$	-	4.317	14.57	29.86	46.11	62.23	79.11	100.7	122.8	168.1	214.3
$cc_{\chi}^{\Phi} \times 10^5$	-	64.85	64.85	64.85	64.85	64.85	64.85	64.85	64.85	64.85	64.85
$\omega_{1j,\chi}^{\Phi}$	-	0.06804	-0.0197	0.1793	-0.02771	0.04568	0.08233	0.862	0.9591	1.097	1.214
$k_{1j,\chi}^{\Phi}$	-	0.008817	0.01369	0.00756	0.01218	0.00687	0.00661	0.0063	0.00618	0.00594	0.0057
$\omega_{ij,\chi}^{\Delta}$	-	-1.93 e-07	5.987 e-06	5.128 e-06	1.507 e-05	1.652 e-05	1.859 e-05	-1.159 e-08	1.805 e-07	1.062 e-06	1.507 e-06
$k_{ij,\chi}^{\Delta}$	-	0.01474	0.00835	0.00756	0.007331	0.00687	0.00661	0.0607	0.00269	0/00342	0.0032
r^2	-	1	1	1	1	1	1	1	1	1	1
SSE	-	2.746 10^{-20}	3.198 10^{-19}	9.187 10^{-15}	1.027 10^{-18}	6.473 10^{-15}	2.527 10^{-15}	4.107 10^{-16}	9.14 10^{-17}	3.761 10^{-14}	2.033 10^{-14}
$RMSE$	-	1.371 10^{-11}	4.68 10^{-11}	7.932 10^{-9}	8.387 10^{-11}	6.658 10^{-9}	4.16 10^{-9}	1.677 10^{-9}	7.916 10^{-10}	5.075 10^{-10}	3.731 10^{-8}

Table S5.

$C_{Y_{0,II}}^{\lambda_{irr},T,2}(t) \times 10^5\ M$	0	0.604	2	4	6.04	8	10	12.5	15	20	25
Reaction I $(\chi = I)$											
$-RK\!: r_{0,X}^{\lambda_{irr},T,2} \times 10^7$	2.43740	2.33750	2.13091	1.88428	1.679743	1.51773	1.3796	1.2376	1.1198	0.94015	0.894
$-Theo\!: r_{0,X}^{\lambda_{irr},T} \times 10^7$	2.44	2.34	2.13	1.88	1.68	1.52	1.38	1.24	1.12	0.940	0.890
$-Fit\!: r_{0,X}^{\lambda_{irr},T,2} \times 10^7$	2.437	2.4518	2.1298	1.9654	1.7446	1.5655	1.3799	1.2167	1.1396	0.95452	0.8420
$\%\ Error$	0.12	4.78	0.01	4.55	3.85	3.00	0.01	1.87	1.76	1.55	5.39
Reaction II $(\chi = II)$											
$-RK\!: r_{0,X}^{\lambda_{irr},T,2} \times 10^7$	-	0.14050	0.42412	0.75007	1.009656	1.20831	1.3729	1.5383	1.6716	1.87121	2.0138
$-Theo\!: r_{0,X}^{\lambda_{irr},T} \times 10^7$	-	0.141	0.424	0.750	1.01	1.21	1.37	1.54	1.67	1.87	2.01
$-Fit\!: r_{0,X}^{\lambda_{irr},T,2} \times 10^7$	-	0.14050	0.42423	0.76962	1.00973	1.22330	1.38310	1.5361	1.67410	1.87242	2.0145
$\%\ Error$	-	0.001	0.025	2.607	0.008	1.241	0.743	0.138	0.149	0.065	0.035

Section 3b. *Traces evolution*

The rate equation of the reactant of *Reaction I* under monochromatic irradiation is worked out from Eq.4, as

$$r_{X_I}^{\lambda_{irr},T,n_{mix}}(t) = \frac{dC_{X_I}^{\lambda_{irr},T,n_{mix}}(t)}{dt} = -\,\Phi_{X_I \to Y_I}^{\lambda_{irr}}\, P_{a_{X_I}}^{\lambda_{irr}}(t) + \,\Phi_{Y_I \to X_I}^{\lambda_{irr}}\, P_{a_{Y_I}}^{\lambda_{irr}}(t) \qquad \textbf{(S5)}$$

By introducing the expression of the absorbed light (Eq.2), we have

$$r_{X_I}^{\lambda_{irr},T,n_{mix}}(t) = -\,\Phi_{X_I \to Y_I}^{\lambda_{irr}}\, A_{X_I}^{\lambda_{irr},T,n_{mix}}(t)\, P_0^{\lambda_{irr}}\, PKF^{\lambda_{irr},T,n_{mix}}(t)$$

$$+\Phi_{Y_I\to X_I}^{\lambda_{irr}}\ A_{Y_I}^{\lambda_{irr},T,n_{mix}}(t)\ P_0^{\lambda_{irr}}\ PKF^{\lambda_{irr},T,n_{mix}}(t) \qquad (\mathbf{S6})$$

Expanding the terms gives

$$r_{X_I}^{\lambda_{irr},T,n_{mix}}(t) = \left[l_{irr}\ P_0^{\lambda_{irr}}\ \left(-\Phi_{X_I\to Y_I}^{\lambda_{irr}}\ \varepsilon_{X_I}^{\lambda_{irr},T,n_{mix}} + \Phi_{Y_I\to X_I}^{\lambda_{irr}}\ \varepsilon_{Y_I}^{\lambda_{irr},T,n_{mix}}\right)\right]\ C_{X_I}^{335}(t)\ \ PKF^{\lambda_{irr},T,n_{mix}}(t)$$

$$+\left[\Phi_{Y_I\to X_I}^{\lambda_{irr}}\ \varepsilon_{Y_I}^{\lambda_{irr},T,n_{mix}}\ l_{irr}\ P_0^{\lambda_{irr}}\ C_{X_I}^{335}(0)\right]\ PKF^{\lambda_{irr},T,n_{mix}}(t) \qquad (\mathbf{S7})$$

or by setting the constants in the square brackets to δ_1 and δ_2, we derive the expression of the rate of reactant I at t_α, as

$$r_{X_I}^{\lambda_{irr},T,n_{mix}}(t_\alpha) = \left(\delta_1\ C_{X_I}^{335}(t_\alpha) + \delta_2\right)\ PKF^{\lambda_{irr},T,n_{mix}}(t_\alpha) \qquad (\mathbf{S8})$$

On one hand we know that when $C_{X_{II}}^{335}(0)$ increases, the positive value of $PKF^{\lambda_{irr},T,n_{mix}}(t_\alpha)$ decreases.

Since the concentration of X_I decreases with reaction time, $r_{X_I}^{\lambda_{irr},T,n_{mix}}(t)$ is always negative or zero (but never positive). The presence of the other absorbing components of the mixture (other than X_I), will shield X_I of a fraction of the incident light and hence the transformation of X_I is hindered, and hence, smaller $C_{X_I}^{335}(t_\alpha)$ will be present in the mixture as $C_{X_{II}}^{335}(0)$ goes higher. As a result, the factor $\left(\delta_1\ C_{X_I}^{335}(t_\alpha) + \delta_2\right)$ will be smaller. Therefore, $r_{X_I}^{\lambda_{irr},T,n_{mix}}(t_\alpha)$ has smaller values when $C_{X_{II}}^{335}(0)$ increases. This means that reactivity of X_I is slowed down with the concentration of its partner, $C_{X_{II}}^{335}(0)$, increases.

Such a behaviour is illustrated in Figure S2.

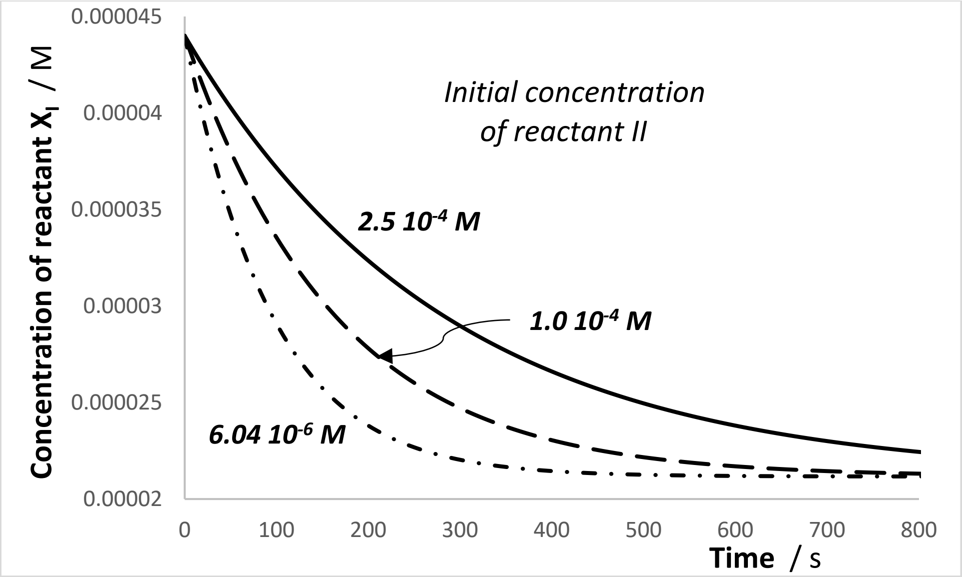

Figure S2.

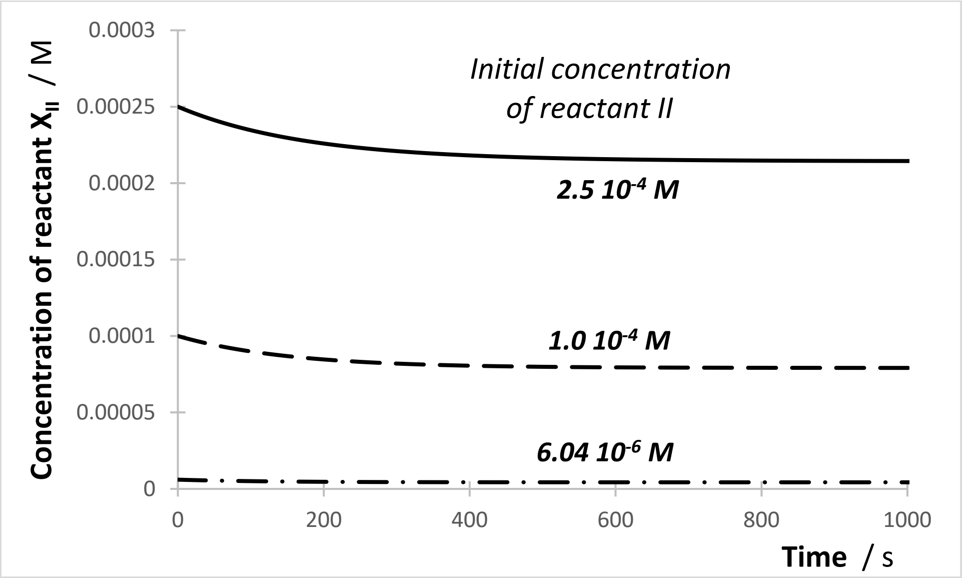

Figure S3.

The expression of the half-life time of Reaction I ($t^I_{\frac{1}{2}}$), where $C^{\lambda_{irr},T,2}_{Y_{j,\chi}}(t) = 0.5\left(C^{\lambda_{irr},T,2}_{Y_{j,\chi}}(0) - C^{\lambda_{irr},T,2}_{Y_{j,\chi}}(\infty)\right)$ since this reaction reaches a pss, can be derived from Eq. S3, as

$$t^I_{\frac{1}{2}} = \frac{-1}{k^{\Phi}_{ij,\chi}}\, ln\left(\left(10^{\frac{0.5\,(C^{\lambda_{irr},T,2}_{Y_{j,\chi}}(0)-C^{\lambda_{irr},T,2}_{Y_{j,\chi}}(\infty))-\omega^{\infty}_{j,\chi}}{\omega^{\Phi}_{ij,\chi}}} - 1\right)\frac{1}{cc^{\Phi}_{j,\chi}}\right) \quad (\mathbf{S9})$$

The half-life time of Reaction II,

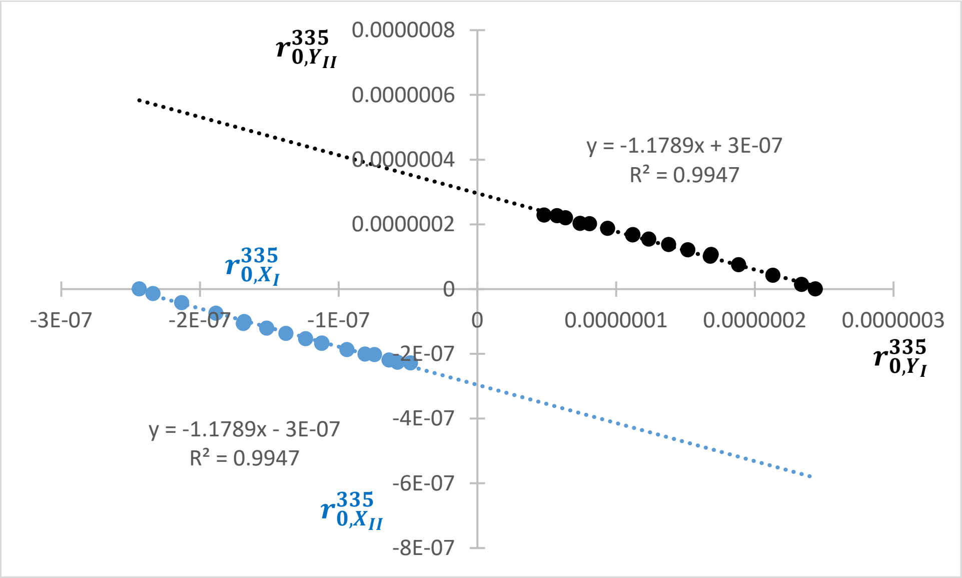

Figure S4.

Section S3c. *Effect of the initial concentration on* $r_{0,X}$, k_r *and* $t_{\frac{1}{2}}$

- *Reaction I*

The variation of the initial reactant when the concentration varies.

Let us consider some simple cases.

a/ the single primary reaction ($X \rightarrow Y$) under isosbestic irradiation (at λ_{isos}): in this case the photokinetics obeys mono-exponential trend as thermal reactions do. The integrated rate-law for the reactant has the general form:

$$C_X^{\lambda_{isos},T,1}(t) = C_X^{\lambda_{isos},T,1}(0) + \frac{r_{0,X}^{\lambda_{isos},T,1}}{k_r^{\lambda_{isos},T,1}}(1 - e^{-k_r^{\lambda_{isos},T,1}\,t}) \qquad (\mathbf{S10})$$

with rate-constant and the initial rate are:

$$k_r^{\lambda_{isos},T,1} = \Phi_{X\rightarrow Y}^{\lambda_{isos}}\,\varepsilon_X^{\lambda_{isos}}\,P_0^{\lambda_{isos}}\,l_{irr}\,PKF^{\lambda_{isos}} \qquad (\mathbf{S11})$$

$$r_{0,X}^{\lambda_{isos},T,1} = -\,k_r^{\lambda_{isos},T,1}\,C_X^{\lambda_{isos},T,1}(0) \qquad (\mathbf{S12})$$

These features clearly indicate that the value of $-\,r_{0,X}^{\lambda_{isos},T,1}$ (Eq. S11), increases with increasing $C_X^{\lambda_{isos},T,1}(0)$. In the same conditions, $k_r^{\lambda_{isos},T,1}$ varies as $PKF^{\lambda_{isos}}$ (Eq. S10), whose values ($PKF^{\lambda_{isos}}$) get smaller with increasing $C_X^{\lambda_{isos},T,1}(0)$ but in a non-linear fashion. Therefore, the half-life time value is deemed to increase with increasing initial concentration (Eq. S13). This is compatible with the smaller values predicted for of reaction rate-constant, $k_r^{\lambda_{isos},T,1}$.

$$t_{\frac{1}{2}}^{\lambda_{isos},T,1} = \frac{ln\,2}{k_r^{\lambda_{isos},T,1}} \qquad (\mathbf{S13})$$

Accordingly, for this reaction, the increase of the initial concentration results in higher initial reactant rates ($-\,r_{0,X}^{\lambda_{isos},T,1}$), a slower reactivity ($k_r^{\lambda_{isos},T,1}$), and longer half-life times ($t_{\frac{1}{2}}^{\lambda_{isos},T,1}$).

b/ the single primary reaction ($X \rightarrow Y$) under non-isosbestic irradiation (at λ_{n-isos}) with $\varepsilon_Y^{\lambda_{n-isos}} = 0$: here in the reaction obeys Φ-order photokinetics. The integrated rate-law for the reactant is:

$$C_X^{\lambda_{n-isos},T,1}(t) = \frac{1}{\varepsilon_X^{\lambda_{n-isos}}\,l_{irr}}\,Log\left(1 + \left(10^{\varepsilon_X^{\lambda_{n-isos}}\,l_{irr}\,C_X^{\lambda_{isos},T,1}(0)} - 1\right)\,e^{-k_r^{\lambda_{n-isos},T,1}\,t}\right) \qquad (\mathbf{S14})$$

and

$$k_r^{\lambda_{n-isos},T,1} = \Phi_{X\rightarrow Y}^{\lambda_{n-isos}}(t)\,\varepsilon_X^{\lambda_{n-isos}}\,P_0^{\lambda_{n-isos}}\,l_{irr}\,ln(10) \qquad (\mathbf{S15})$$

$$r_{0,X}^{\lambda_{n-isos},T,1} = -\,\frac{k_r^{\lambda_{n-isos},T,1}}{\varepsilon_X^{\lambda_{n-isos}}\,l_{irr}\,ln(10)}\left(1 - 10^{-\,\varepsilon_X^{\lambda_{n-isos}}\,l_{irr}\,C_X^{\lambda_{isos},T,1}(0)}\right) \qquad (\mathbf{S16})$$

For this reaction case, the rate-constant is independent of the reactant initial concentration (Eq. S15), the initial reactant rate has the same variation with initial concentration as the non-linear term of Eq. S16, i.e., $\left(1 - 10^{-\,\varepsilon_X^{\lambda_{n-isos}}\,l_{irr}\,C_X^{\lambda_{isos},T,1}(0)}\right)$, which increase with $C_X^{\lambda_{isos},T,1}(0)$ (i.e., the values of $-\,r_{0,X}^{\lambda_{n-isos},T,1}$

increase). Finally, the logarithm in the equation of the half-life time (SI-Eq. S17) increases with increasing values of $C_X^{\lambda_{isos},T,1}(0)$.

$$t_{\frac{1}{2}}^{\lambda_{n-isos},T,1} = \frac{1}{k_r^{\lambda_{n-isos},T,1}}\ ln\left(\frac{10^{0.5\,\varepsilon_X^{\lambda_{n-isos}}\,l_{irr}\,C_X^{\lambda_{isos},T,1}(0)} - 1}{10^{\varepsilon_X^{\lambda_{n-isos}}\,l_{irr}\,C_X^{\lambda_{isos},T,1}(0)} - 1}\right) \qquad (\mathbf{S17})$$

Hence, for this reaction, the increase of the initial concentration results in higher initial reactant rates ($-\,r_{0,X}^{\lambda_{isos},T,1}$), a constant reactivity ($k_r^{\lambda_{n-isos},T,1}$), and longer half-life times ($t_{\frac{1}{2}}^{\lambda_{n-isos},T,1}$).

c/ the single photoreversible ($X \leftrightharpoons Y$) and primary photoreactions ($X \rightarrow Y$) under non-isosbestic irradiation (at λ_{n-isos}), and with both $\varepsilon_Y^{\lambda_{n-isos}} \neq 0$.

The general Φ-order integrated rate-law for the reactant, obtained from the semi-empirical methd, is:

$$C_X^{\lambda_{n-isos},T,1}(t) = C_X^{\lambda_{n-isos},T,1}(\infty) + \frac{1}{\left(\varepsilon_X^{\lambda_{n-isos}} - \varepsilon_Y^{\lambda_{n-isos}}\right)\,l_{irr}} \times$$

$$Log\left(1 + \left(10^{\left(\varepsilon_X^{\lambda_{n-isos}}-\varepsilon_Y^{\lambda_{n-isos}}\right)\,l_{irr}\,\left(C_X^{\lambda_{isos},T,1}(0)-C_X^{\lambda_{isos},T,1}(\infty)\right)} - 1\right)\,e^{-\,k_r^{\lambda_{n-isos},T,1}\,t}\right) \qquad (\mathbf{S18})$$

with the rate-constant being

$$k_r^{\lambda_{n-isos},T,1} = \left(\Phi_{X\rightarrow Y}^{\lambda_{n-isos}}\,\varepsilon_X^{\lambda_{n-isos}} + \Phi_{Y\rightarrow X}^{\lambda_{n-isos}}\,\varepsilon_Y^{\lambda_{n-isos}}\right)\,P_0^{\lambda_{n-isos}}\,l_{irr}\,PKF^{\lambda_{n-isos}}(\infty) \qquad (\mathbf{S19})$$

and the initial reactant rate

$$r_{0,X}^{\lambda_{n-isos},T,1} = -\,\Phi_{X\rightarrow Y}^{\lambda_{n-isos}}\,\varepsilon_X^{\lambda_{n-isos}}\,P_0^{\lambda_{n-isos}}\,l_{irr}\,\left(1 - 10^{-\,\varepsilon_X^{\lambda_{n-isos}}\,l_{irr}\,C_X^{\lambda_{isos},T,1}(0)}\right) \qquad (\mathbf{S20})$$

Since, at pss ($t \approx \infty$), the concentrations of the composition of the medium increase with $C_X^{\lambda_{isos},T,1}(0)$, and hence, the total absorbance, $A_{tot}^{\lambda_{n-isos}}(\infty) = A_X^{\lambda_{n-isos}}(\infty) + A_Y^{\lambda_{n-isos}}(\infty)$, increases, which induces $PKF^{\lambda_{n-isos}}(\infty)$ to decrease (for higher values of $C_X^{\lambda_{isos},T,1}(0)$). Therefore, $k_r^{\lambda_{n-isos},T,1}$ should decrease when $C_X^{\lambda_{isos},T,1}(0)$ increases, and therefore, $t_{\frac{1}{2}}^{\lambda_{n-isos},T,1}$ should increase (because the reaction is slower). In the same condition, $-\,r_{0,X}^{\lambda_{n-isos},T,1}$ increases as $\left(1 - 10^{-\,\varepsilon_X^{\lambda_{n-isos}}\,l_{irr}\,C_X^{\lambda_{isos},T,1}(0)}\right)$.

So, for these reactions, the increase of the initial concentration results in higher initial reactant rates ($-\,r_{0,X}^{\lambda_{isos},T,1}$), a slower reactivity ($k_r^{\lambda_{n-isos},T,1}$), and longer half-life times ($t_{\frac{1}{2}}^{\lambda_{n-isos},T,1}$). Experimental confirmation os such trends were provided for primary photoprocess whose product absorbs (Maafi and Maafi, 2013) and photoreversible reactions (Maafi and Maafi, 2015a), both under monochromatic/non-isosbestic light. The latter reactions, record an increase of both $-\,r_{0,X}^{\lambda_{isos},T,1}$ and $t_{\frac{1}{2}}^{\lambda_{n-isos},T,1}$, and a decrease of $k_r^{\lambda_{n-isos},T,1}$.

This pattern should hold for any single reaction, when this reaction is not the only absorbing species in the medium. The trend was also observed for many other single photoreactions under either mono- or polychromatic irradiation (Maafi, 2023).

This pattern seems to be conserved when the reaction (whose reactant concentration increase) is performed in a mixture, as demonstrated by the results of the present ms.

- *Reaction II*

The initial reactant-rate for *Reaction II*, when $C_{X_{II}}^{335}(0) \neq 0$, is

$$r_{0,X_{II}}^{\lambda_{irr},T,n_{mix}} = -\,\Phi_{X_{II}\rightarrow Y_{II}}^{\lambda_{irr}}\ \varepsilon_{X_{II}}^{\lambda_{irr},T,n_{mix}}\ l_{irr}\ P_0^{\lambda_{irr}}\ C_{X_{II}}^{335}(0)\ \ PKF^{\lambda_{irr},T,n_{mix}}(0) \qquad \textbf{(S21)}$$

The reduction expected for the value of $PKF^{\lambda_{irr},T,n_{mix}}(0)$ is largely overtaken by the increase of $C_{X_{II}}^{335}(0)$ in the product, so that $-\,r_{0,X_{II}}^{\lambda_{irr},T,n_{mix}}$ effectively increases with $C_{X_{II}}^{335}(0)$. Figure S5 shows the patterns of both $PKF^{\lambda_{irr},T,n_{mix}}(0)$ and the product, $C_{X_{II}}^{335}(0)\ PKF^{\lambda_{irr},T,n_{mix}}(0)$, when $C_{X_{II}}^{335}(0)$ increases, for the binary mixture depicted in Scheme 2 using the data provided in Section 2.7.1.

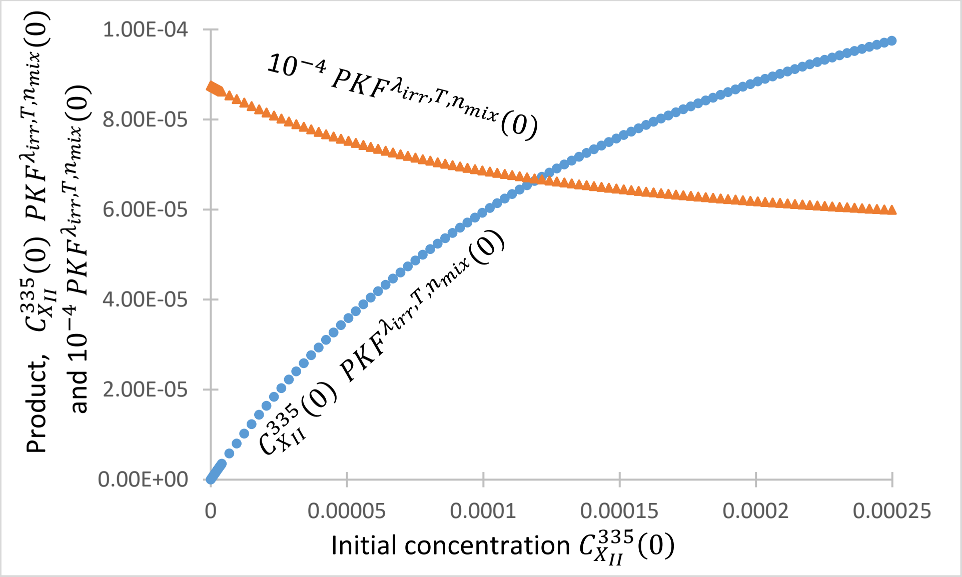

Figure S5.

Section 3d. *Correlation of* $r_{0,X}$ *and* $C_X\ (0)$ *ratios according to Eq.12*

Raw data taken from (Nguyen *et al.*, 2016).

concentration / mM		**rate-constant /h[-1]**	
phenol	o-cresol	**kp**	**kc**
0	0.3		**1**
0.3	0.3	**0.73**	**0.65**
0.45	0.3	**0.5**	**0.44**
0.9	0.3	**0.24**	**0.28**
phenol	4-cp		
0	0.3		**1.66**
0.3	0.3	**0.83**	**1**

0.45	0.3	**0.5**	**0.6**
0.9	0.3	**0.27**	**0.34**

Worked out initial rates and ratios

**Initial rates**			Phenol/o-cresol	Phenol/4-CP
**ro,p**	**ro,c**	cp/cc	ro,p/ro,c	
**0**	**0.3**	0	0	
**0.219**	**0.195**	1	1.123076923	
**0.225**	**0.132**	1.5	1.704545455	
**0.216**	**0.084**	3		
**0**	**0.498**	0		0
**0.249**	**0.3**	1		0.83
**0.225**	**0.18**	1.5		1.25
**0.243**	**0.102**	3		2.38235294

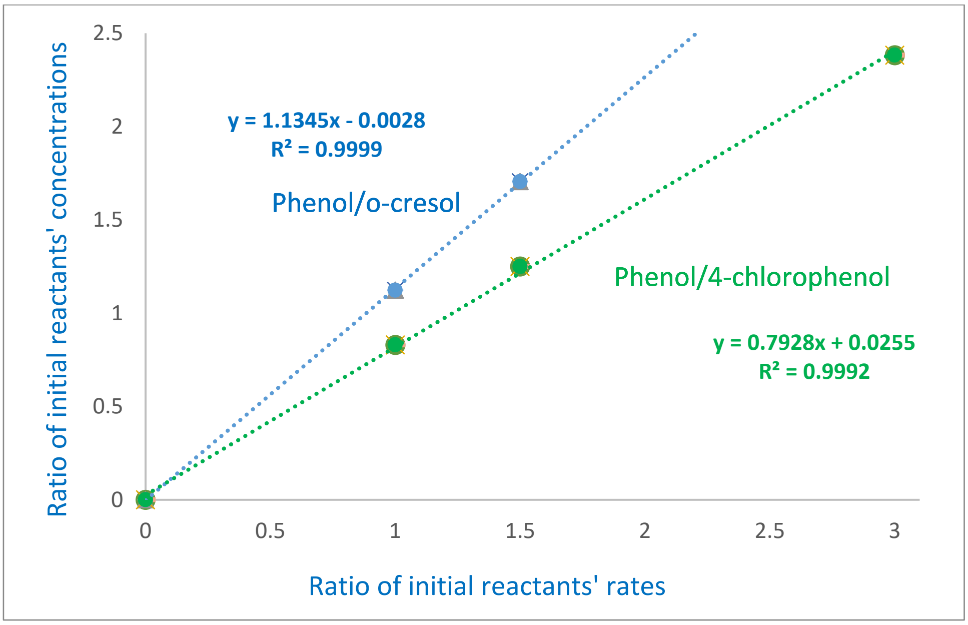

Figure S6.

Section S4. Ternary system under polychromatic light (Scheme 3 in the main text)

Investigation of the effect of the initial concentration of the reactant of *Reaction I* on the initial rates of the three reactants.

The explicit equations used for the fitting of reactant and product of the ternary system, has the general following form.

$$C_{Y_{j,\chi}}^{Lp,\Delta\lambda,T,n_{mix}}(t) = \omega_{j,\chi}^{\infty} + \omega_{1j,\chi}^{\Phi}\, Log\left(1 + cc_{j,\chi}^{\Phi}\, e^{-k_{1j,\chi}^{\Phi}\, t}\right) + \omega_{2j,\chi}^{\Phi}\, Log\left(1 + cc_{j,\chi}^{\Phi}\, e^{-k_{2j,\chi}^{\Phi}\, t}\right)$$

$$+ \omega_{3j,\chi}^{\Phi}\, Log\left(1 + cc_{j,\chi}^{\Phi}\, e^{-k_{3j,\chi}^{\Phi}\, t}\right) + \omega_{1j,\chi}^{\Delta}\, e^{-k_{1j,\chi}^{\Delta}\, t} + \omega_{2j,\chi}^{\Delta}\, e^{-k_{1j,\chi}^{\Delta}\, t} \quad (\mathbf{S22})$$

The absorption coefficient spectra of reactions II and III are shown in Figures S7 and S8.

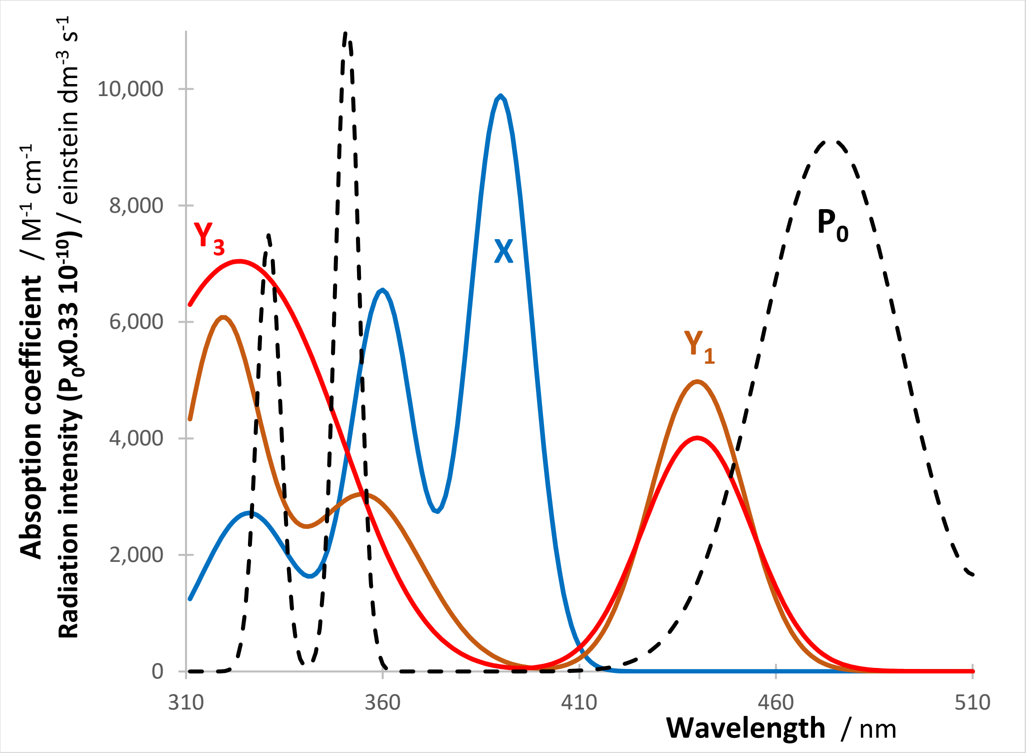

Figure S7: *Reaction II* (Scheme 3)

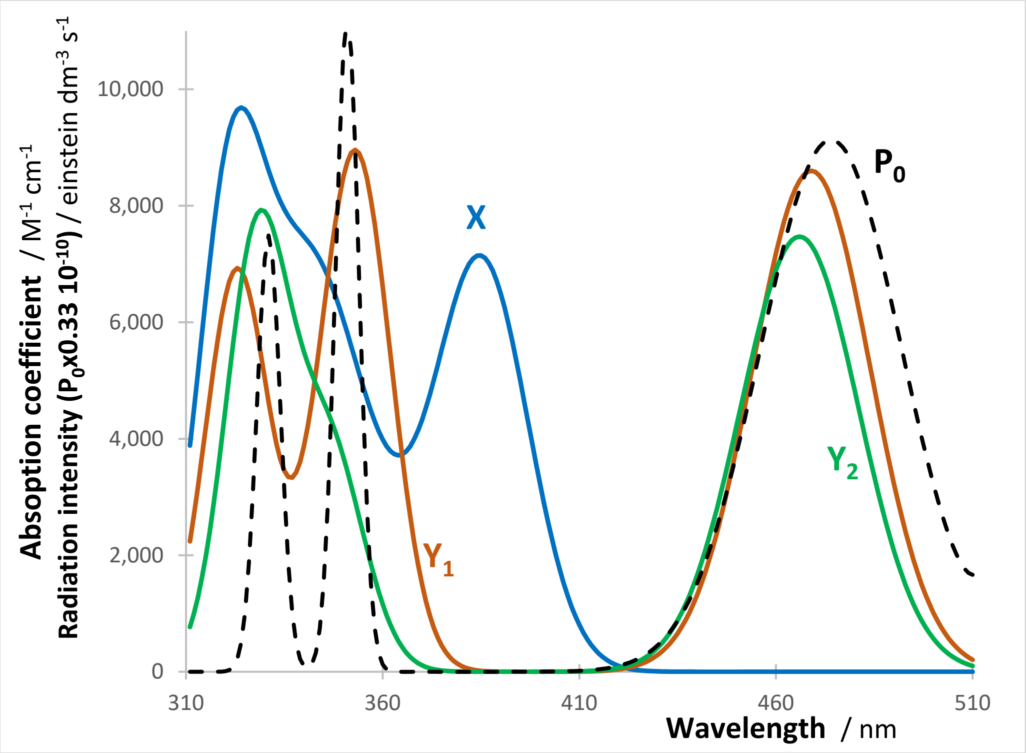

Figure S8: *Reaction III* (Scheme 3)

The variation of the quantum yields with wavelength of the photochemical reaction-steps in reactions II and III are shown in Figures S9 and S10.

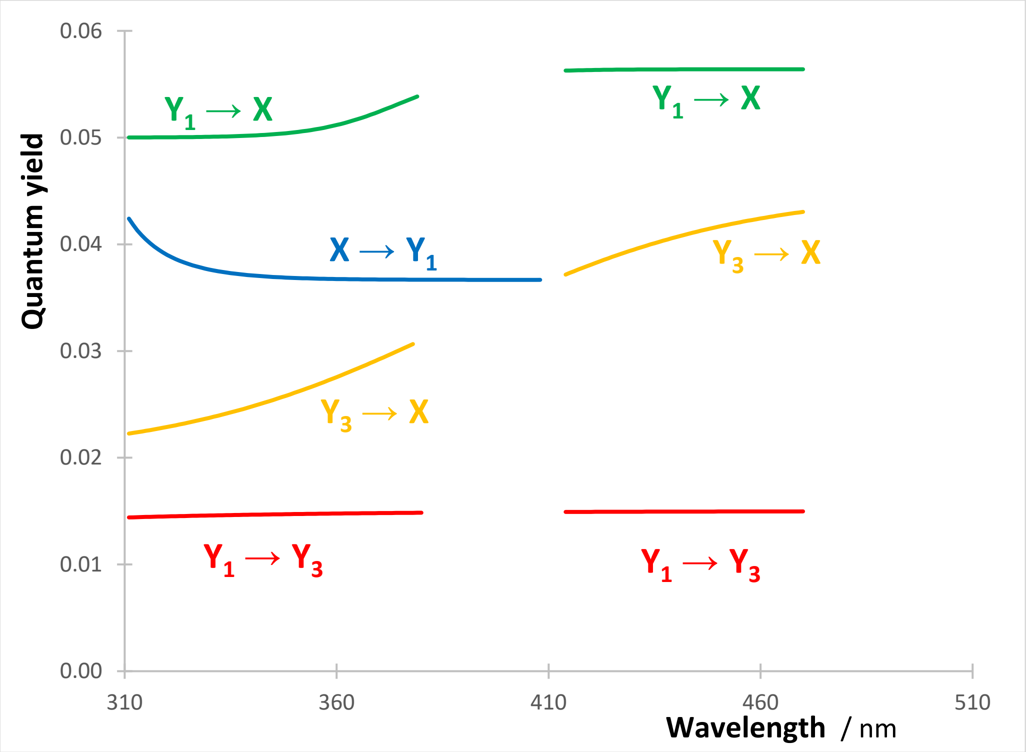

Figure S9: *Reaction II* (Scheme 3). With $k^{\Delta}_{Y_{1,II} \to X_{II}} = 0.001\ s^{-1}$, $k^{\Delta}_{Y_{3,II} \to X_{II}} = 0.0174\ s^{-1}$, $k^{\Delta}_{Y_{1,II} \to Y_{3,II}} = 0.0201\ s^{-1}$, and $k^{\Delta}_{Y_{3,II} \to Y_{1,II}} = 0.0095\ s^{-1}$

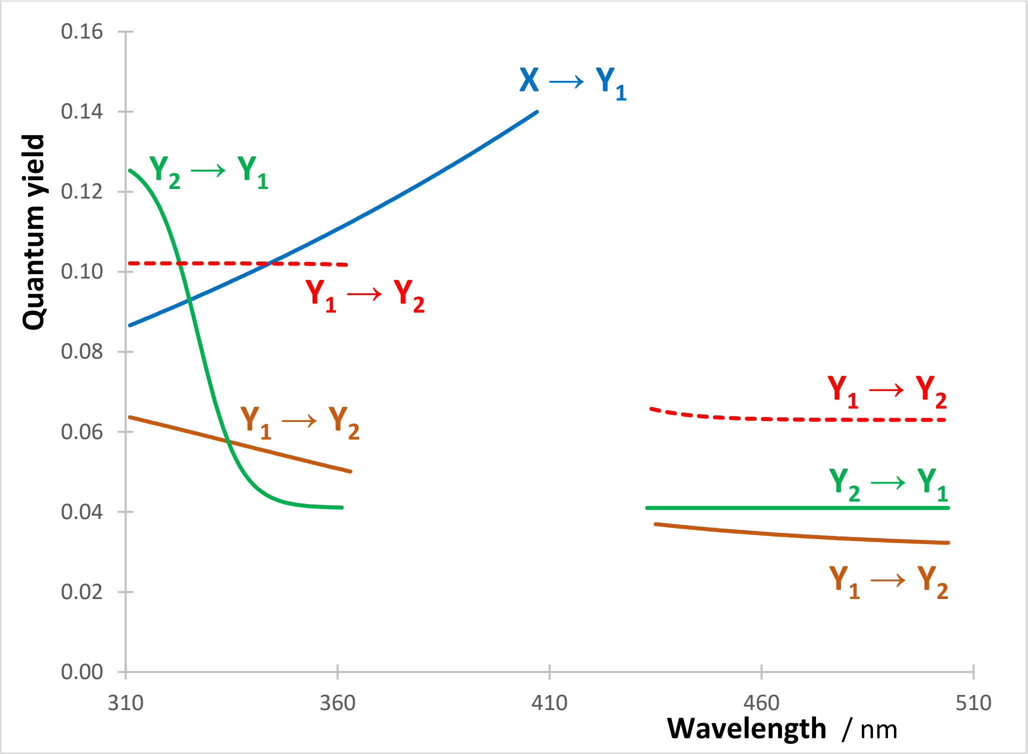

Figure S10: *Reaction III* (Scheme 3)

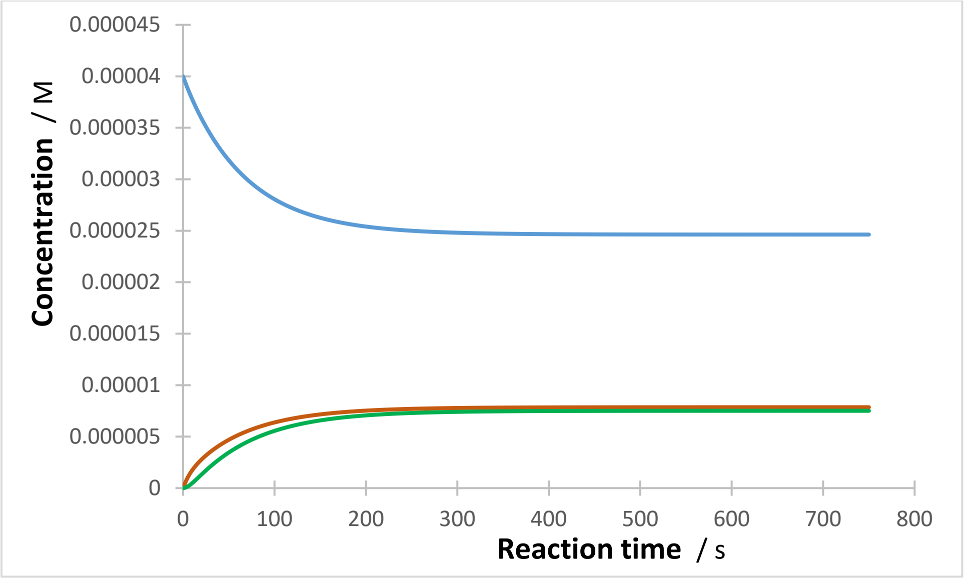

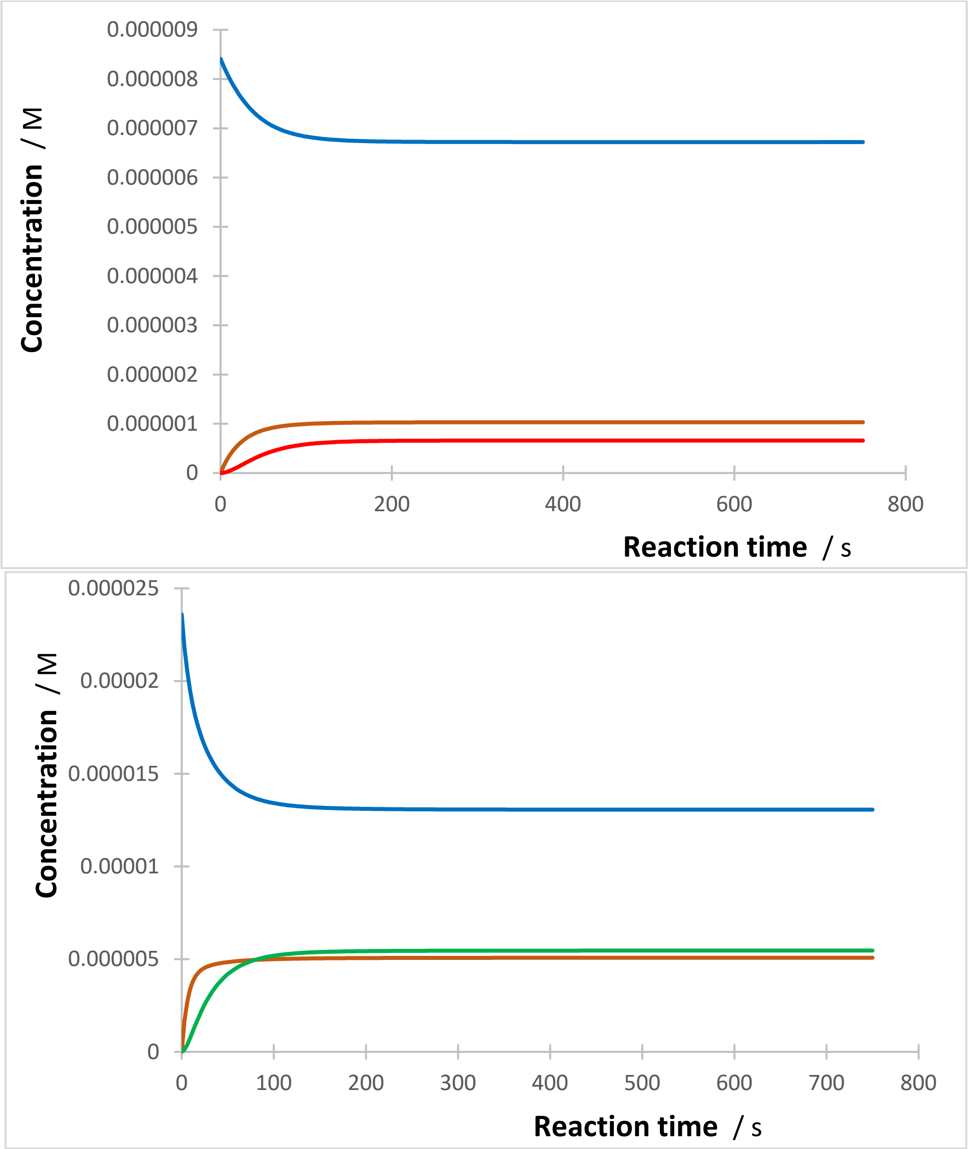

Figure S11: Examples of concentration traces of the three reactions of the mixture, from top: *Reactions I* ($C_{X,I}(0) = 4\ 10^{-5}$ M), *II* ($C_{X,II}(0) = 8.41\ 10^{-6}$ M) and *III* ($C_{X,III}(0) = 2.36\ 10^{-5}$ M)

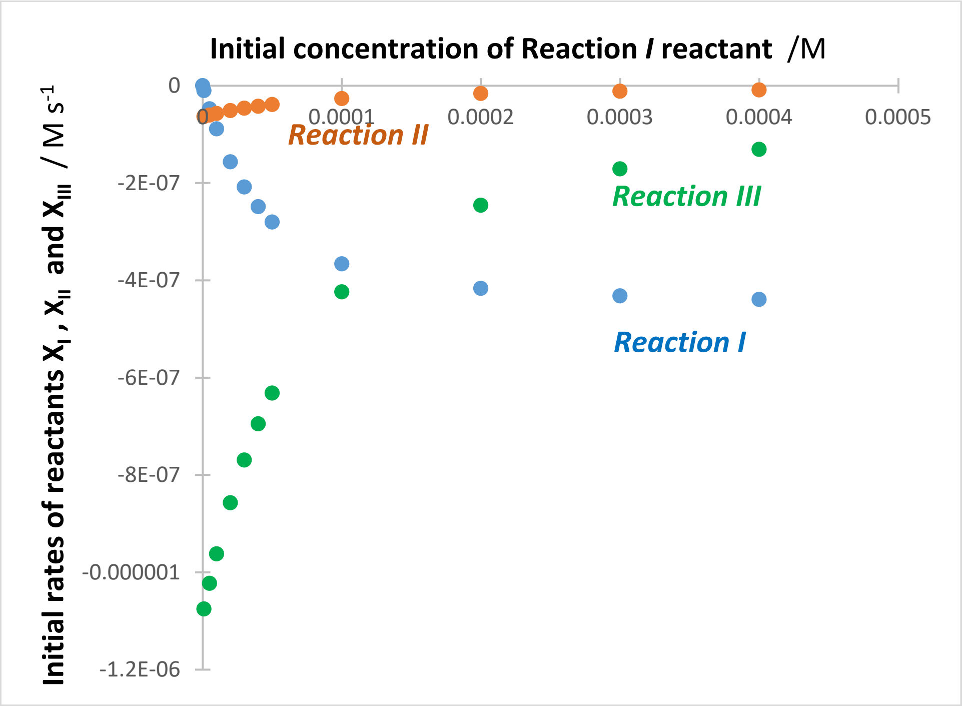

Figure S12.

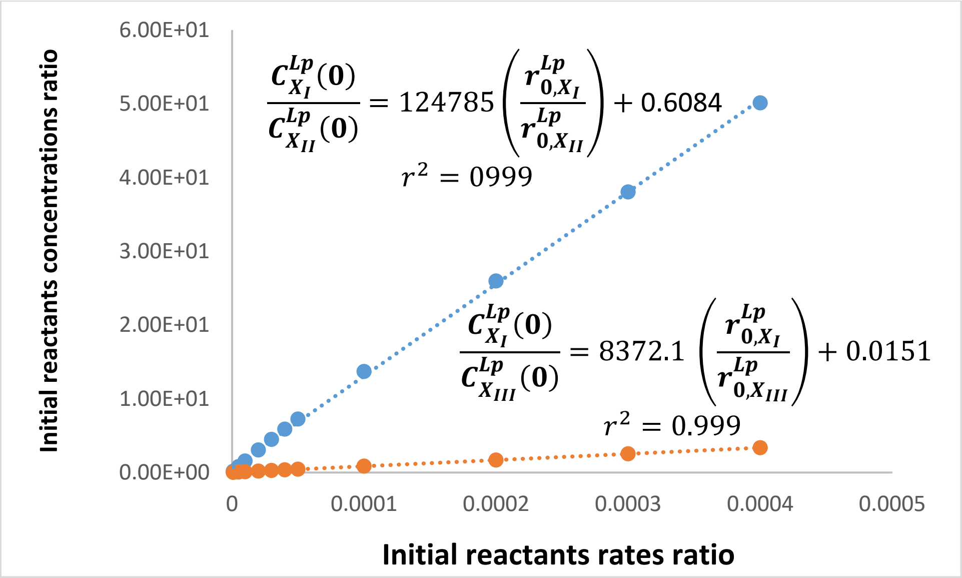

Figure S13.

$$Theo{:}\, r_{0,X_\chi}^{Lp,\Delta\lambda,T,n_{mix}} = -\sum_{\lambda_{irr}=\lambda_a}^{\lambda_b}\left(\sum_{j';\, j'\neq 0}^{n_{\Phi,0,\chi}} \Phi_{X_\chi\to Y_{j',\chi}}^{\lambda_{irr}}\; P_{a_{Y_{0,\chi}}}^{\lambda_{irr}}(0)\right)$$

$$= -\sum_{\lambda_{irr}=\lambda_a}^{\lambda_b}\left(\sum_{j';\, j'\neq 0}^{n_{\Phi,0,\chi}} \Phi_{X_\chi\to Y_{j',\chi}}^{\lambda_{irr}}\; A_{Y_{j,\chi\ or\ j',\chi}}^{\lambda_{irr},T,n_{mix}}(0)\; P_0^{\lambda_{irr}}\; PKF^{\lambda_{irr},T,n_{mix}}(t)\right)$$

$$= -\,C_{X_\chi}^{\lambda_{irr},T,n_{mix}}(0) \sum_{\lambda_{irr}=\lambda_a}^{\lambda_b}\left(\sum_{j';\, j'\neq 0}^{n_{\Phi,0,\chi}} \Phi_{X_\chi\to Y_{j',\chi}}^{\lambda_{irr}}\; \varepsilon_{X_\chi}^{\lambda_{irr}}(0)\; l_{irr}\;\; P_0^{\lambda_{irr}}\; PKF^{\lambda_{irr},T,n_{mix}}(0)\right) \qquad \textbf{(S23)}$$

$$\frac{r_{0,X_I}^{Lp,\Delta\lambda,T,n_{mix}}}{r_{0,X_{II}}^{Lp,\Delta\lambda,T,n_{mix}}} = \left[\frac{\sum_{\lambda_{irr}=\lambda_a}^{\lambda_b}\left(\sum_{j';\,j'\neq 0}^{n_{\Phi,0,\chi}} \Phi_{X_I\rightarrow Y_{j',I}}^{\lambda_{irr}}\ \varepsilon_{X_I}^{\lambda_{irr},n_{mix}}(0)\ l_{irr}\ \ P_0^{\lambda_{irr}}\ PKF^{\lambda_{irr},T,n_{mix}}(0)\right)}{\sum_{\lambda_{irr}=\lambda_a}^{\lambda_b}\left(\sum_{j';\,j'\neq 0}^{n_{\Phi,0,\chi}} \Phi_{X_{II}\rightarrow Y_{j',II}}^{\lambda_{irr}}\ \varepsilon_{X_{II}}^{\lambda_{irr},n_{mix}}(0)\ l_{irr}\ \ P_0^{\lambda_{irr}}\ PKF^{\lambda_{irr},T,n_{mix}}(0)\right)}\right] \frac{C_{X_I}^{\lambda_{irr},T,n_{mix}}(0)}{C_{X_{II}}^{\lambda_{irr},T,n_{mix}}(0)}$$

$$(\mathbf{S24})$$

and

$$\frac{r_{0,X_I}^{Lp,\Delta\lambda,T,n_{mix}}}{r_{0,X_{III}}^{Lp,\Delta\lambda,T,n_{mix}}} = \left[\frac{\sum_{\lambda_{irr}=\lambda_a}^{\lambda_b}\left(\sum_{j';\,j'\neq 0}^{n_{\Phi,0,\chi}} \Phi_{X_I\rightarrow Y_{j',I}}^{\lambda_{irr}}\ \varepsilon_{X_I}^{\lambda_{irr},n_{mix}}(0)\ l_{irr}\ \ P_0^{\lambda_{irr}}\ PKF^{\lambda_{irr},T,n_{mix}}(0)\right)}{\sum_{\lambda_{irr}=\lambda_a}^{\lambda_b}\left(\sum_{j';\,j'\neq 0}^{n_{\Phi,0,\chi}} \Phi_{X_{II}\rightarrow Y_{j',III}}^{\lambda_{irr}}\ \varepsilon_{X_{III}}^{\lambda_{irr},n_{mix}}(0)\ l_{irr}\ \ P_0^{\lambda_{irr}}\ PKF^{\lambda_{irr},T,n_{mix}}(0)\right)}\right] \frac{C_{X_I}^{\lambda_{irr},T,n_{mix}}(0)}{C_{X_{III}}^{\lambda_{irr},T,n_{mix}}(0)}$$

$$(\mathbf{S25})$$

Which is a linear relationship between the ratios:

$$\frac{r_{0,X_I}^{Lp,\Delta\lambda,T,n_{mix}}}{r_{0,X_{III}}^{Lp,\Delta\lambda,T,n_{mix}}} = Constant\ \times\ \frac{C_{X_I}^{\lambda_{irr},T,n_{mix}}(0)}{C_{X_{III}}^{\lambda_{irr},T,n_{mix}}(0)}$$

$$(\mathbf{S26})$$
